# Pretransplant Systemic Therapies for Hepatocellular Carcinoma: A Systematic Review of Posttransplant Outcomes

**DOI:** 10.1007/s12029-026-01399-w

**Published:** 2026-02-14

**Authors:** Joana Rodrigues Ribeiro, Célia Procter, Christian Toso

**Affiliations:** 1 Abdominal Surgery Division, Department of Surgery, University Hospitals of Geneva, Geneva, Switzerland; 2 Epidemiology, Biostatistics and Prevention Institute, University of Zurich, Zurich, Switzerland; 3 Faculty of Medicine, University of Geneva, Geneva, Switzerland

**Keywords:** Hepatocellular carcinoma (HCC), Liver transplantation (LT), Neoadjuvant, Systemic treatment, Protein kinase inhibitors (PKIs), Immune checkpoint inhibitors (ICIs)

## Abstract

**Purpose:**

Systemic therapies are increasingly being explored as strategies to downstage or bridge patients with unresectable hepatocellular cancer (HCC) to liver transplantation (LT). This systematic review aimed to assess pooled survival and posttransplant outcomes in patients receiving such therapies.

**Methods:**

We searched PubMed, Embase and Medline through September 2025 to identify interventional and observational studies on adult patients with HCC treated with any systemic regimen before LT. Outcomes included 1- and 3-year overall survival (OS) and proportions of recurrence, mortality and rejection. We assessed risk of bias and quality using established tools and presented pooled cumulative incidence proportions and prevalences in forest plots.

**Results:**

Fourteen studies were included, encompassing 412 patients, with up to 26 possible overlaps. After LT, the pooled OS was 88% at 1 year (95%CI: 81% to 93%) and 76% at 3 years (95%CI: 65% to 85%). Acute rejection occurred in 21% patients overall (95%CI: 12% to 34%) and mortality in 17% (95%CI: 10% to 26%). HCC recurrence concerned 23% of patients (95%CI: 16% to 32%) at a median time-to-recurrence of 13.5 months (95%CI: 4.7 to 22.4).

**Conclusion:**

We pooled posttransplant cumulative incidence proportions in HCC patients who received systemic therapy before LT, providing reference estimates to help interpret outcomes across HCC stages.

**Supplementary Information:**

The online version contains supplementary material available at https://doi.org/10.1007/s12029-026-01399-w.

## Introduction

### Guideline-Based Treatments

Hepatocellular carcinoma (HCC), the third leading cause of cancer-related deaths [1], constitutes the indication for approximately 30% of liver transplantations (LT) in Europe and the USA [2]. 

According to the Barcelona Clinic Liver Cancer (BCLC) classification [3–5], curative options are mainly indicated for patients with very early to early-stage diseases and include liver resection (LR) and transplantation. For intermediate stages (BCLC-B), locoregional therapies (LRT) are the standard of care and, although not curative, may downstage the disease sufficiently to enable subsequent curative options such as LR or LT. Systemic therapies are primarily indicated for advanced stages (BCLC-C) – notably immune checkpoint inhibitors (ICIs) and tyrosine kinase inhibitors (TKIs) – and in selected BCLC-B patients who are unsuitable for, or progress after LRT. This has the practical consequence that systematic options may lead to a downstaged disease with curative options. Nonetheless, the use of PKIs and ICIs as formal downstaging therapies towards curative treatments remains undefined in the official algorithm and is not addressed for BCLC-C patients.

### Systemic Treatments across Years

Prior to the era of immunotherapy and targeted molecules, a few trials tested the efficacy of systemic traditional chemotherapy for HCC. The main molecules tested were Doxorubicin, 5-Fluorouracil, Gemcitabine, and Irinotecan, but none proved to be effective [6]. Thus, systemic chemotherapy is no longer indicated for HCC and is currently only used as last resort therapy in selected patients.

In 2007, the SHARP trial results on the efficacy of Sorafenib in the treatment of HCC showed improved overall survival and reduced tumor progression, enabling its use as first-line treatment for advanced, unresectable HCC in Europe and the USA [7]. Since then, other protein kinase inhibitors (PKIs) have been introduced, including Lenvatinib, Regorafenib, and Cabozantinib – the last two being 2nd-line options at the moment [4]. 

More recently, immunotherapy has taken the forefront in HCC treatment. Following the IMBrave150 trial in 2020 [8] and HIMALAYA trial in 2022 [9], Atezolizumab and Bevacizumab as well as Durvalumab and Tremelimumab are currently the standard of care for ICIs in BCLC-C HCC [4]. Ongoing trials are assessing other molecules, as well as their combinations with LRT, such as the CheckMate 74 W trial, which is testing the combination of Nivolumab and Ipilimumab with TACE [10]. Nonetheless, concerns about the safety of immunotherapy prior to LT have been raised. Probably due to their intrinsic immunogenic properties, they may increase the propensity to rejection reactions. Some cohort studies have reported graft loss in the most severe cases [11–16]. Safety concerns regarding this lack of guidance on ICIs prior to liver transplantation have recently been addressed by Moeckli et al., who concluded that a washout period of more than 50 days before LT is safe with respect to the risk of rejection [17]. 

While individual trials of PKIs and ICIs have reported favorable findings, establishing pooled outcome data for systemic therapies is critical to generate standard reference values for posttransplant prognosis.

### Rationale and Aims

The current body of literature lacks synthesis on posttransplant outcomes in patients with HCC who received systemic therapies as bridging or downstaging strategies to meet transplant criteria. Our review aims to address this gap by (1) synthetizing the body of literature by determining cumulative incidence proportions such as overall and recurrence-free survival (OS and RFS), and proportions of rejection, recurrence and mortality in patients with HCC who received traditional chemotherapy, ICIs and/or PKIs as bridging or downstaging tools to liver transplant and (2) identifying areas of improvement in the methodology of further trials.

## Methods

### Protocol and Registration

This review was prospectively registered with the International Prospective Register of Systematic Reviews (PROSPERO) under reference ID CRD420251073869, after confirming no review on the same topic was being conducted. Original and updated versions of the protocol are available on PROSPERO. The present study was written in accordance with the Preferred Reporting Items for Systematic Reviews and Meta-Analysis (PRISMA) guidelines [18] (see Online Resource 1), and the Cochrane Handbook was consulted where applicable [19]. No ethical approval was required for this review article.

### Eligibility Criteria

#### Population

The population of interest included adult patients diagnosed with unresectable HCC who received any systemic therapy prior to LT (see Table 1). This targeted population encompassed three main groups: (1) patients with early-stage disease on the waiting list within transplant criteria such as Milan [21] and University of California San Francisco (UCSF) [22] who received systemic therapy as a bridging strategy to prevent HCC progression; (2) patients with intermediate-stage (BCLC-B) disease who were successfully downstaged with systemic therapy and became eligible for LT; and (3) patients with advanced-stage (BCLC-C or even D) disease who responded favorably to PKIs and/or ICIs, achieving sufficient tumor regression to meet transplant criteria. Notably, this last group fell outside the standard BCLC treatment algorithm. We included patients regardless of prior HCC treatment, including LR. Although recurrence after LR was initially planned as an exclusion criterion, many studies included such patients or did not report prior treatments and excluding them would have substantially reduced the study pool. Moreover, excluding recurrence after LR but not after ablation was inconsistent and would have resulted in a less representative population of LT candidates; therefore, inclusion criteria were revised accordingly. Nonetheless, we excluded patients with recurrent HCC after LT, as well as those with atypical HCC subtypes, such as fibrolamellar HCC, mixed HCC-choriocarcinoma, or HCC with features of pediatric cancers, such as hepatoblastoma.

**Table 1:** Summary table of eligibility criteria with PICOS elements [20]

	Inclusion criteria	Exclusion criteria
**P – Population**	Adult patients Unresectable HCC Previous HCC treatment: none, LRT and/or LR	Age < 18 years Prior LT Atypical HCC subtypes: fibrolamellar HCC, mixed HCC-cholangiocarcinoma, mixed HCC-hepatoblastoma
**I – Intervention**	Any systemic therapy prior to LT (in monotherapy or combination)	–
**C – Control**	No intervention, placebo, standard of care (e.g., LRT)	–
**O – Outcomes**	Primary: 1- and 3-year OS and RFS, TTR, proportion of recurrence and mortality Secondary: proportion of ACR, graft loss and complications CDC ≥ IIIa, postoperative LOS	
**S – Study design**	RCTs, nRCTs, 1-arm and 2-arm cohort studies	Descriptive studies (case reports, short case-series), conference papers, isolated abstracts, posters, reviews, meta-analyses

**Abbreviations**: ACR, Acute cellular rejection; CDC, Clavien-Dindo Classification; HCC, Hepatocellular carcinoma; LOS, Length of stay; LR, Liver resection; LRT, Locoregional therapy; LT, Liver transplant; nRCT, non-Randomized controlled trial; OS, Overall survival; RFS, Recurrence-free survival; RCT, Randomized controlled trial; TTR, Time-to-recurrence

#### Intervention and Control

Interventions consisted of any therapy administered systemically before LT, such as chemotherapy, immunotherapy (including ICIs) and PKIs (including TKIs). Such therapies could either be used in monotherapy or combined with other systemic or locoregional therapies. We included any control measure, e.g., no systemic therapy, placebo, or standard of care.

#### Outcomes

Studies had to report at least one of our primary or secondary outcomes. Our primary outcomes were oncological outcomes defined as 1- and 3-year OS and RFS, median time-to-recurrence (TTR) and proportions of recurrence and mortality. Secondary outcomes consisted of surgical outcomes: proportions of rejection, graft loss and postoperative complications with a Clavien-Dindo Classification [23] (CDC) grade ≥ IIIa, as well as median postoperative length of stay (LOS). Other exploratory outcomes included objective response rate (ORR), duration of systemic therapy, washout and posttransplant follow-up. We initially aimed to include safety outcomes, such as adverse events (AEs) related to chemotherapy, PKIs or ICIs. Nonetheless, due to the absence of the official Common Terminology Criteria for Adverse Events (CTCAE) classification [24] in early studies, and to variations in its versions over our long study period, along with heterogeneous AEs reporting and attribution, we did not analyze this outcome.

#### Study Design

We included randomized controlled trials (RCTs), non-RCTs, and cohort studies that analyzed post-LT outcomes in patients with unresectable HCC. Both single-arm and two-arm studies were considered for inclusion. We excluded only descriptive studies, reviews, meta-analyses, conference papers, isolated abstracts and posters from our analysis.

### Search Strategy

A systematic literature search was performed on June 7, 2025, using PubMed, MEDLINE (via Ovid) and EMBASE (via Elsevier) databases, with Boolean combinations of keywords, as detailed in Online Resource 3. We did not apply any time restrictions. Only full articles written in a language understood by at least one reviewer were included (English, French, German, Spanish and Portuguese). Finally, we also conducted a manual search of the references of selected articles and systematic reviews. A final search was conducted across all three databases on September 10.

### Selection Process

Articles were saved on Zotero^®^ [25], exported in an Excel^®^ Table [26], and duplicates were excluded. Two independent reviewers (JRR, CP), blinded to each other’s decision, performed an initial screening of eligible articles based on titles and abstracts, and discrepancies between individual judgements were resolved after discussion between both reviewers and consultation with a third party (CT) if no consensus was reached.

While screening retrieved articles, we identified overlapping cases through a reference search of cohort studies, with particular attention to hospitals and the time intervals. To avoid direct overlap in data analysis while optimizing the quality of the selected studies, we decided to exclude from the primary analysis cohort studies and trials that were included in larger cohort studies. When studies with similar designs had overlapping patient populations, we included the most comprehensive and extracted the non-overlapping cases from the excluded study, depending on their designs. In two situations, we could not distinguish the non-overlapping cases and had to keep all studies which concerned a maximum of 26 (9 + 17) patients.

### Data Collection Process

Data collection was also performed by two independent reviewers (JRR, CP) in Excel^®^ tables, and discrepancies between individual judgements were resolved after discussion between both reviewers and consultation with a third party (CT). Data were extracted as reported; no imputation was performed for missing values, and study investigators were not contacted.

Besides our primary, secondary, and exploratory endpoints, we exported each study title, first author name, country, year of publication, study period and design, number of patients in the intervention (± control) group, systemic treatment name and type (i.e., chemotherapy, ICIs, PKIs, and combined PKI-ICI).

We extracted sample size and/or proportions for discrete variables. We chose to favor medians and interquartile ranges (IQRs) for continuous variables, as our cohorts were relatively small and therefore at risk of a more skewed distribution. For studies reporting minimal, maximal, and either median or mean values, we estimated IQRs (± median) using Wan et al.’s method [27, 28] (see Online Resource 2). All data underwent final verification prior to statistical analysis. Each version of both selection and collection processes was thoroughly recorded with RStudio^®^ and saved through GitHub^®^ Desktop [29]. 

### Data Definition

#### Acute Cellular Rejection

While we originally wanted to analyze all rejection reactions – acute, subacute and chronic – all studies reported acute cellular rejection (ACR) reactions, mostly within 30 postoperative days, but none documented subacute or chronic rejection reactions. We therefore only analyzed patients with ACR. Diagnostic approaches for ACR varied across studies, encompassing biochemical markers and histological examination, with criteria ranging from nonspecific biological perturbations post-LT to biopsy-proven rejection.

#### Overall and Recurrence-Free Survival Versus Mortality and Recurrence Proportions

OS and RFS values were estimated in the original studies using Kaplan-Meier curves and extracted at 1- and 3-year posttransplant. These cumulative incidence proportions should be distinguished from recurrence and mortality proportions, which were reported as event proportions at last follow-up.

#### Objective Response Rate

Most studies assessed radiological response before LT according to the modified Response Evaluation Criteria In Solid Tumors (mRECIST) criteria [27], which are commonly described as the most accurate [28, 29]; we included in our database the RECIST version used in each study [30, 31]. 

### Risk of Bias and Quality Assessment

Risk of bias and quality were assessed using the Cochrane RoB 2.0 tool [30] for RCTs and the ROBINS-I tool [31] for non-randomized studies, including non-RCTs and comparative cohort studies. We used the Joanna Briggs Institute (JBI) Critical Appraisal tool [32] to assess risk of bias and quality in non-comparative cohort studies. All studies were classified according to Hartling et al.’s classification tool [33] for adequate assessment, and summary visualizations were generated using the online *robvis* tool [34]. Assessment of each study was performed by one reviewer (JRR) at two different time points (before and after data analyses); the results were consistent.

### Statistical Analyses

We reported descriptive statistics as medians with interquartile ranges (IQRs) for continuous variables, and as proportions (%) with absolute counts for categorical variables.

Pooled analyses were performed using random-effects models with maximum likelihood estimation of between-study variance. We pooled cumulative incidence proportions and proportions using inverse-variance weighting with a logit transformation and a 0.5 continuity correction for zero events. We pooled continuous outcomes reported as medians with IQRs using the McGrath method [35–37]. Forest plots were generated using the *meta* [38] and *metamedian* [39] packages, as well as the *I*^*2*^ statistics, *T*^*2*^ and Cochran’s Q *X*^*2*^ test to assess heterogeneity. Subgroup pooled analyses were performed according to the neoadjuvant regimen (traditional chemotherapy vs. PKI vs. ICI vs. combined PKI-ICI) to explore potential differences in pooled estimates and to present outcomes without formal comparisons. Due to limited data, a fixed-effect model was used to pool subgroup pooled estimates. As we chose to display forest plots derived from random-effects models, the pooled subgroup-specific estimates we reported in the results are not shown graphically but are available as supplementary material. Color coding was used to distinguish studies by intervention (i.e., the neoadjuvant regimen), allowing quick visual assessment of potential heterogeneity and possible trends across study subtypes. Sensitivity analyses were performed when a subgroup’s pooled estimate appeared to fall outside the 95% confidence interval (95%CI) of other subgroups. Formal comparisons between the original and post–sensitivity analysis overall pooled estimates were conducted using Z-tests. Reporting bias was not assessed because the pooled proportion analysis lacked effect sizes with standard errors. The certainty of evidence was not assessed.

We performed all statistical analyses in the R statistical programming environment [40, 41] and used the *ggplot2* package for all visualization plots [42]. For all analyses, a two-tailed probability of type I error of ≤ 0.05 was considered statistically significant.

## Results

### Study Selection

**Fig. 1:**
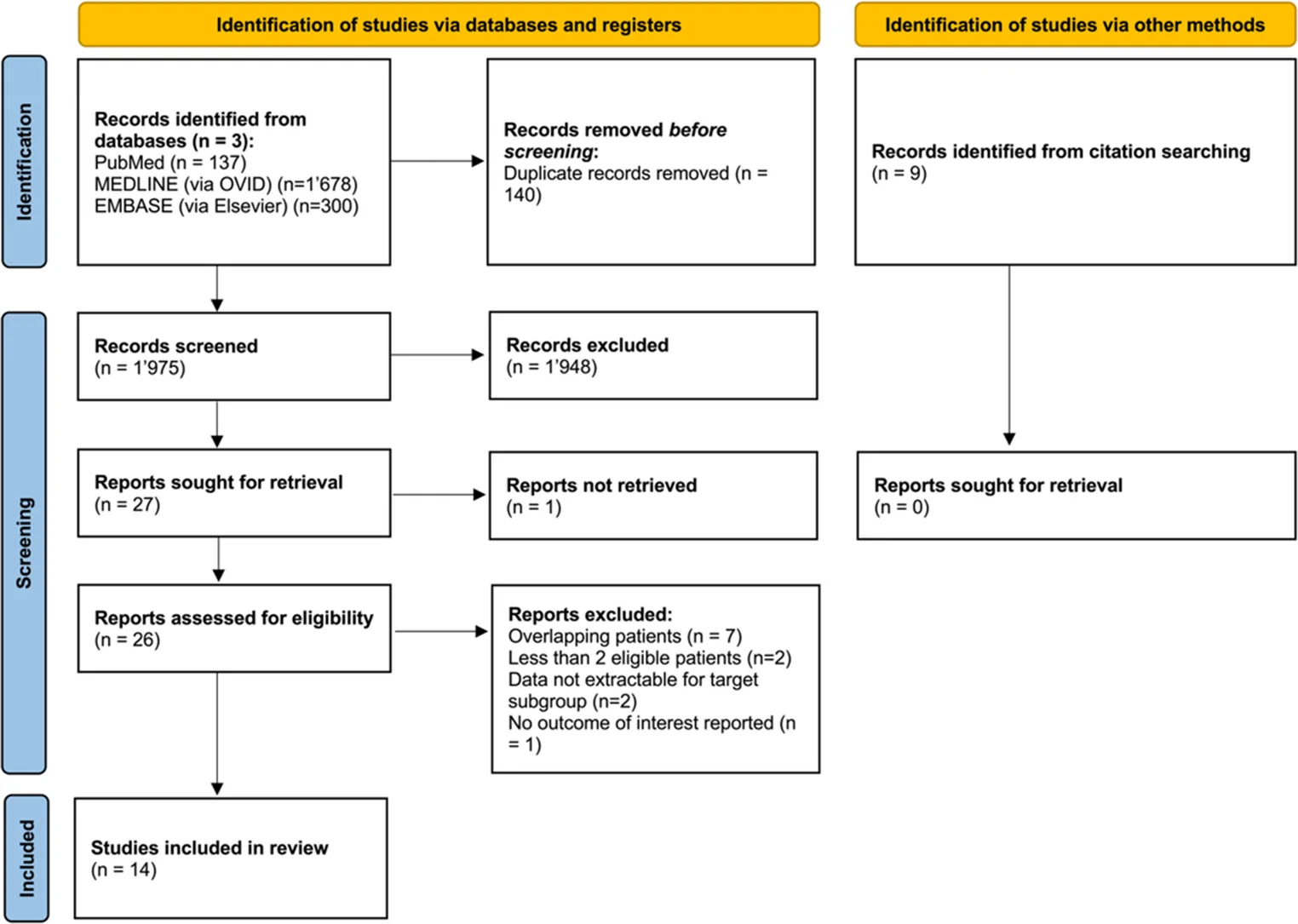
**Flowchart for study and patient inclusion**. Literature search yielded a total of 2’124 articles, including 9 through citation searching. After sequential screening and assessment processes, 14 studies were included in the final review. The flowchart was based on the PRISMA 2020 template [18]. This work is licensed under CC BY 4.0. To view a copy of this license, visithttps://creativecommons.org/licenses/by/4.0/

A literature search identified 1’975 records after removing duplicates, of which 1’948 did not meet eligibility criteria. Of the 26 reports retrieved and assessed, seven were excluded due to overlapping cases, two included only one eligible patient, two lacked data on eligible patients, and one did not report outcomes of interest. In parallel, reference lists from retrieved reports were also assessed – nine in total – from which none was selected. Finally, our literature research yielded a total of 14 studies [17, 43–55], as represented in the PRISMA flowchart (see Fig. 1) [18].

### Study Characteristics

**Fig. 2:**
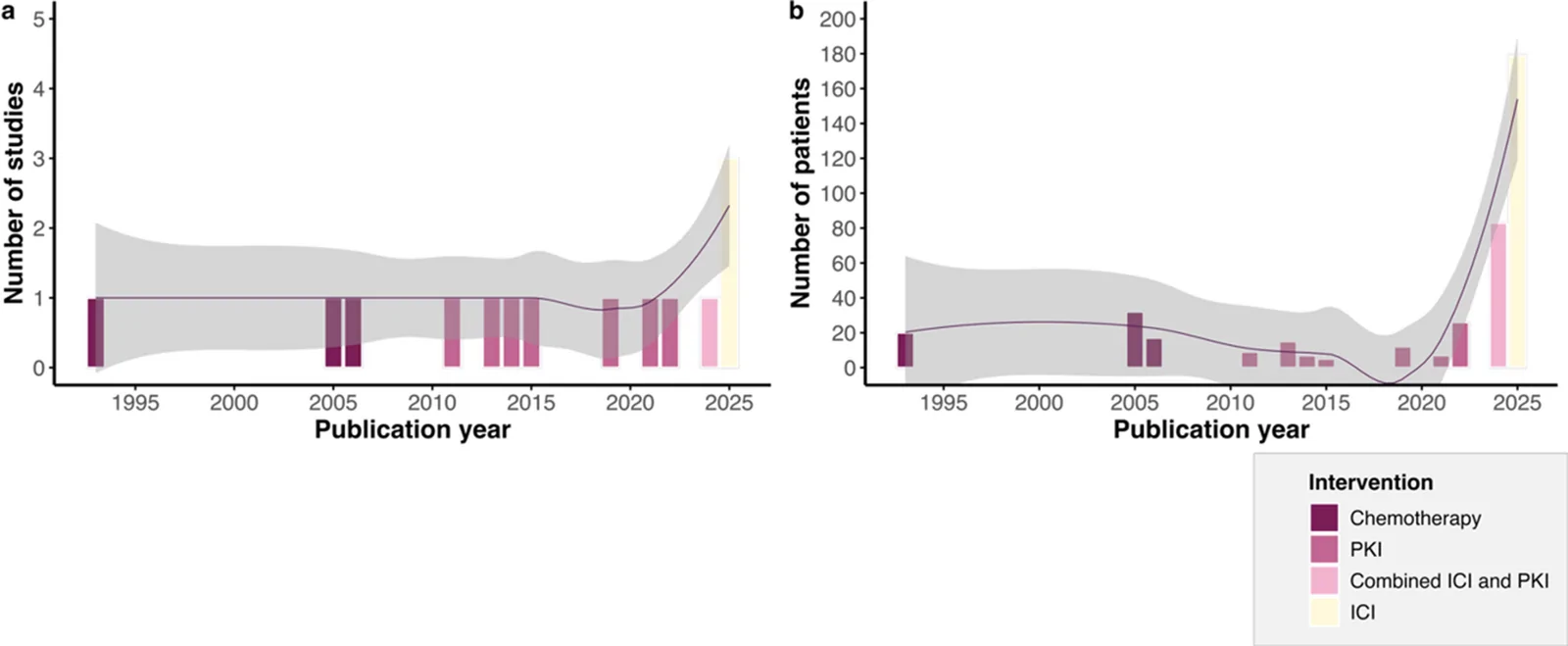
Studies on neoadjuvant systemic therapies for hepatocellular carcinoma before liver transplant. Graphical representations displaying an increase of the number of studies (a) and included patients (b) published every year, particularly those receiving ICIs. Those curves are based on selected studies. Other overlapping cohorts were excluded from separate plotting to avoid double-counting participants within therapy-specific analyses. Studies and patients are grouped by intervention*, i.e*., neoadjuvant therapy allocated (chemotherapy vs. PKI vs. combined PKI-ICI vs. ICI) with distinct colors. Statistical analysis: Data are presented as histograms, with locally estimated scatterplot smoothing (LOESS) lines with 95%CI added to visualize the trends. Abbreviations: ICI, Immune checkpoint inhibitor; nRCT, non-Randomized controlled trial; PKI, Protein kinase inhibitor; RCT, Randomized controlled trial.

The included studies were published from 1993 to 2025 and involved between 386 and 412 patients. The number of studies published and the number of patients enrolled increased over time, suggesting growing clinical and research interest in this area, particularly in ICIs (see Fig. 2).

Cohort studies accounted for most included studies (64.3% =9/14) and enrolled patients (83.0% =342/412). RCTs and non-RCTs contributed to 61 and nine patients across four and one studies, respectively (see Table 2). Besides two international cohorts including 33.0% of total patients (*n* = 136), studies from the USA enrolled the largest proportion of patients (22.8%) and were the most represented in the review (5/14).

**Table 2:** Descriptive table of included studies

Author	Publication year	Journal	Design	Comparative design	Treatment type	Treatment strategy	Included patients	Country
Pokorny et al.	2005	Am J Transplant	RCT	Comparative	Chemotherapy	Bridging	32	Austria
Söderdahl et al.	2006	Transpl. Int.	RCT	Comparative	Chemotherapy	Bridging	17	Sweden, Finland
Kulik et al.	2014	J. Hepatol.	RCT	Comparative	PKI	Bridging	7	USA
Hoffmann et al.	2015	BMC Cancer	RCT	Comparative	PKI	–	5	Germany
Truesdale et al.	2011	Transpl. Int.	nRCT	Comparative	PKI	Downstaging	9	USA
Stone et al.	1993	Gastroenterology	Cohort study	Non-comparative	Chemotherapy	–	20	USA
Frenette et al.	2013	Transpl. Int.	Cohort study	Comparative	PKI	Bridging	15	USA
Eilard et al.	2019	BMC Cancer	Cohort study	Non-comparative	PKI	Bridging	12	Sweden
Woei-A-Jin et al.	2021	Oncologist	Cohort study	Non-comparative	PKI	Downstaging	7	Netherlands
Minoux et al.	2022	Transpl. Int.	Cohort study	Non-comparative	PKI	Both	26	France
Guo et al.	2024	Am J Transplant	Cohort study	Non-comparative	Combined ICI and PKI	Both	83	China
Moeckli et al.	2025	J. Hepatol.	Cohort study	Non-comparative	ICI	Both	119	more than 3 countries
Tabrizian et al.	2025	J. Hepatol.	Cohort study	Non-comparative	ICI	Both	43	USA
Tabrizian* et al.	2025	JHEP Rep.	Cohort study	Non-comparative	ICI	Downstaging	17	more than 3 countries

* We used (*) to distinguish Tabrizian et al.’s study involving non-American centers from the VITALY study, also written by Tabrizian et al.

Abbreviations: ICI, Immune checkpoint inhibitor; nRCT, non-Randomized controlled trial; PKI, Protein kinase inhibitor; RCT, Randomized controlled trial; USA, United States of America

Half of the studies investigated PKIs (7/14), accounting for 19.7% of all patients (*n* = 81). In contrast, ICIs were represented in 21.4% of studies, including 43.4% of all patients (*n* = 179). Patients receiving combined PKI-ICI accounted for 20.1% of patients (*n* = 83) enrolled in a single study. Chemotherapy was investigated in three studies, the oldest in our pool, and represented 16.7% of all patients (*n* = 69).

Among PKIs, 91.4% of patients were treated with Sorafenib in monotherapy, whereas Lenvatinib was the agent most frequently combined with ICIs (60.2% =50/83). Within the large spectrum of ICIs, most patients on ICI monotherapy received Nivolumab, Pembrolizumab, and the combined Atezolizumab-Bevacizumab, and the most frequently used ICI in combination with PKIs was Camrelizumab (37.3% =31/83). Doxorubicin was used in all chemotherapy studies. Combined regimens of systemic and locoregional therapies were always used, except in studies on chemotherapy. Whilst different LRT modalities were reported across and within studies, the most frequently reported was TACE (*n* = 9).

Most studies included patients with both downstaging and bridging strategies (65.8% =271/412), and patients had heterogeneous initial disease stages, ranging from BCLC-A to D. Nonetheless, regardless of their initial stage and systemic therapy strategy, all patients eventually met transplant criteria.

### Risk of Bias and Quality Assessment

**Fig. 3:**
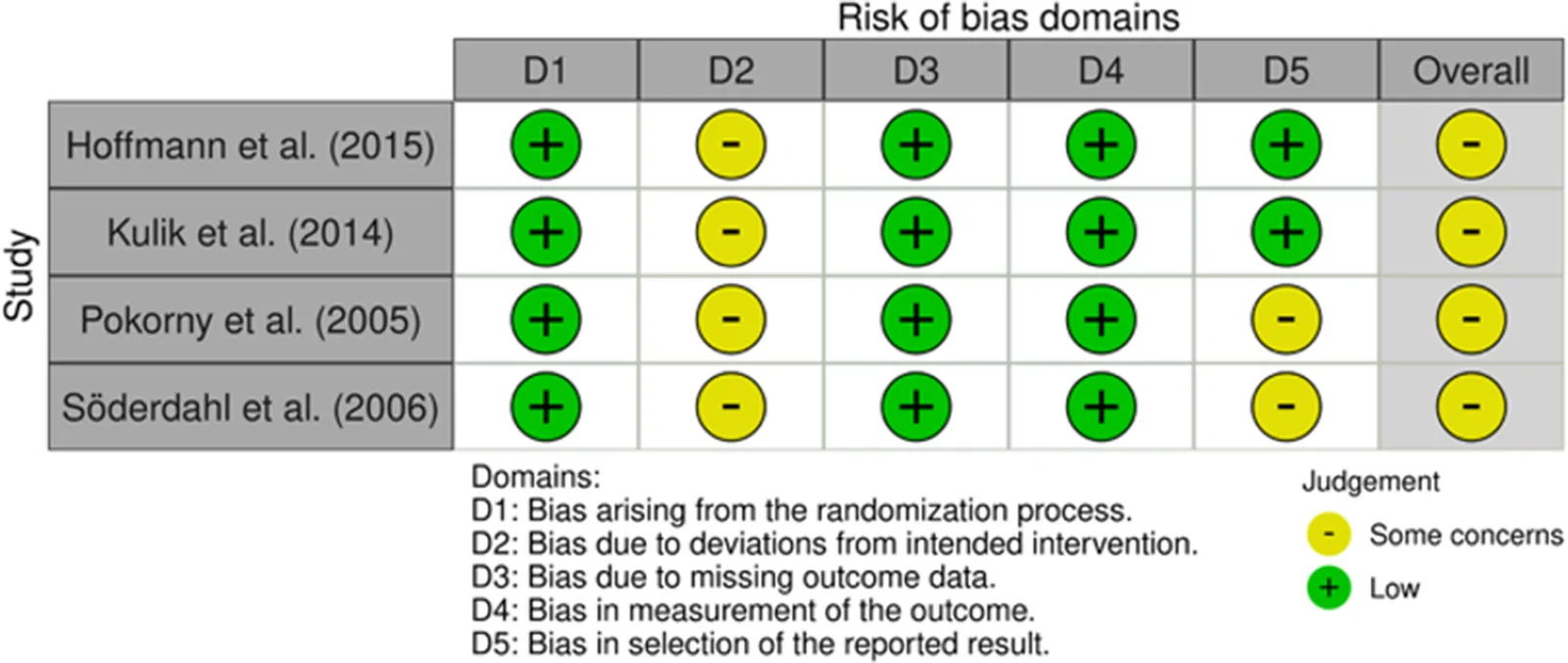
Randomized controlled trials’ risk of bias assessment through RoB 2.0 tool. Assessment of the four RCTs showed some concerns for risk of bias, according to the five-domain RoB 2.0 tool. The figure was generated with *robvis* tool [33].

**Fig. 4:**
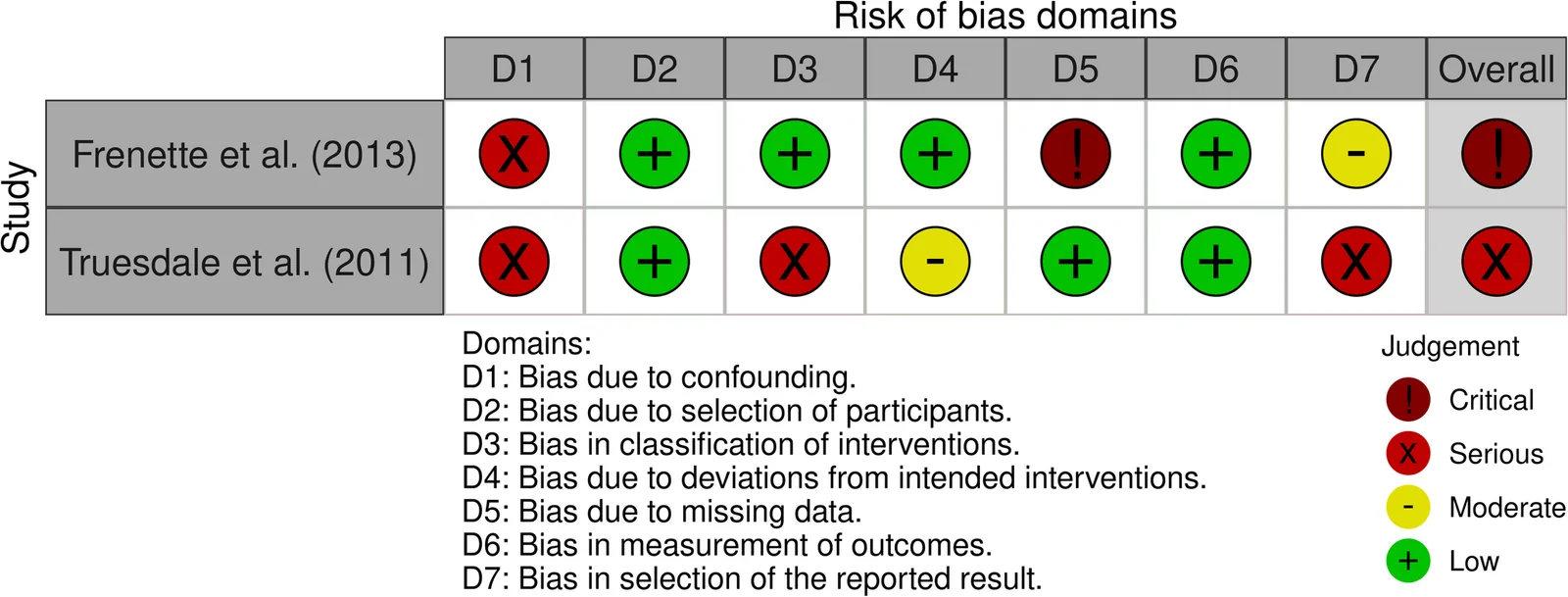
Non-randomized studies’ risk of bias assessment through the ROBINS-I tool. All studies were considered at critical or serious risk of bias, according to the seven-domain ROBINS-I tool. In accordance with most reviews publicly available, ROBINS-I domain-level assessment was fully conducted for studies, even when a critical risk of bias was determined at the preliminary stage. The pre-assessment qualified both studies at critical risk of bias because of the absence of confounding control. The figure was generated with *robvis* tool [34]

**Fig. 5:**
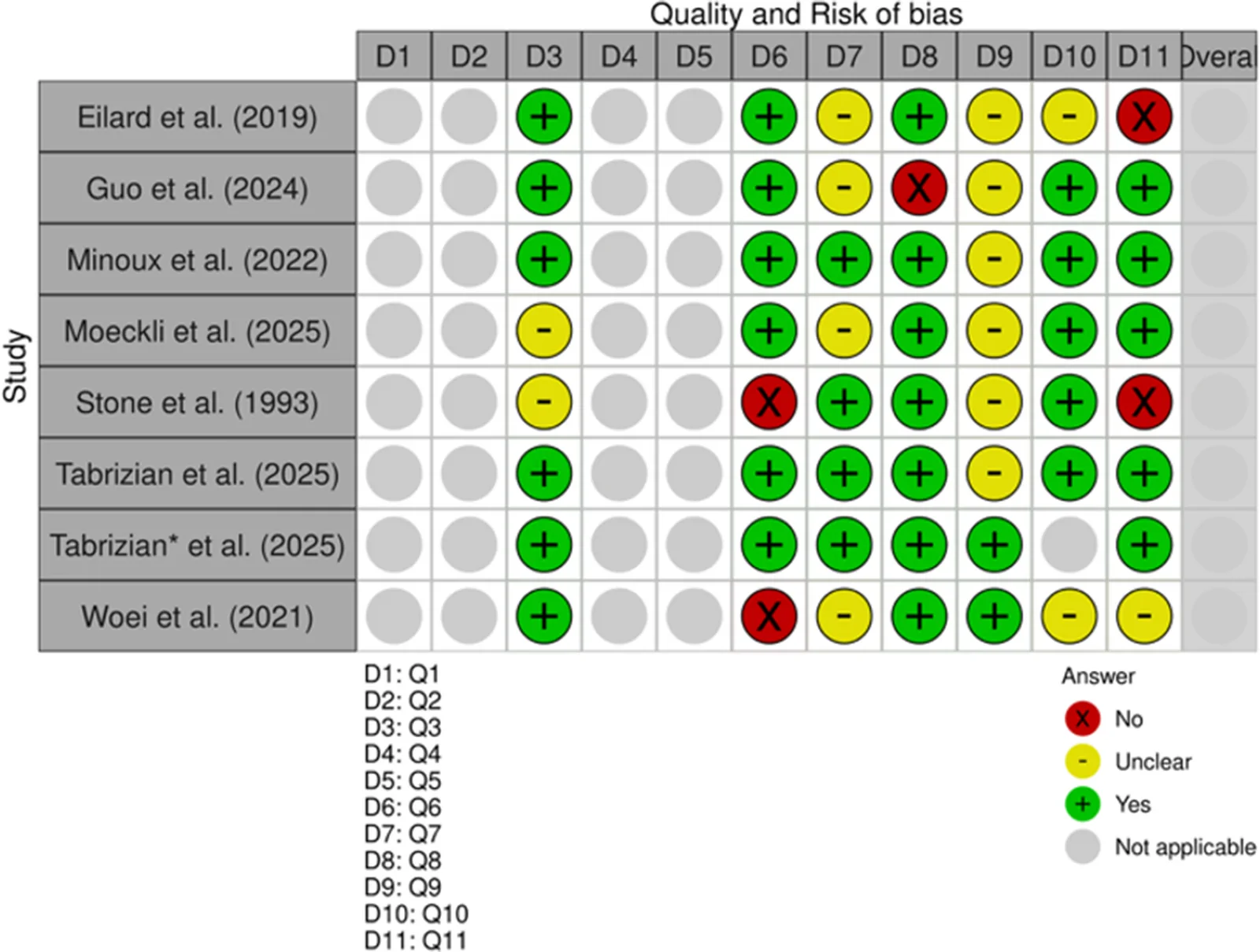
Cohort studies’ quality and risk of bias assessment through the JBI Critical Appraisal tool. JBI (Joanna Briggs Institute) Critical Appraisal tool for cohort studies has been used to assess risk of bias and quality. All studies assessed were composed of non-comparative single-arm cohorts; therefore, questions 1–2 and 4–5 were answered “Not applicable”. The figure was generated with *robvis* tool [34]

Summary tables of the results of the individual risk of bias and quality assessment are displayed in Figs. 3, 4 and 5. All four RCTs were judged to have some concerns about risk of bias due to deviations from the intended interventions; 50.0% also had concerns about selection bias in the reported results. Both non-randomized studies were considered at serious and critical risk of bias, as key confounders were not controlled for. Additionally, one showed serious bias in participant selection and selective reporting; the other had significant missing data. The cohort study assessment was not applicable to questions 1–2 and 4–5, as they were single-arm studies. Most studies did not clarify whether follow-up was complete; two used inadequate statistical analyses; and two identified, only intraoperatively, that patients did not fulfill inclusion criteria due to advanced-stage HCC.

### Results of Syntheses

Pooled median therapy duration, reported in ten studies, was 3.8 months (95%CI: 2.78 to 4.83, *I*^*2*^ = 81.7%), and the median washout, pooled from seven studies, was 53.3 days (95%CI: 27.7 to 78.9, *I*^*2*^ = 93.3%) for any regimen and 56.5 days (95%CI: 47.3 to 65.6, *I*^*2*^ = 0.0%) for ICI specifically. Pooled ORR across five studies was 56% (95%CI: 19% to 87%, *I*^*2*^ = 89.0%), reflecting the high heterogeneity of the enrolled patients, as expected. Pooled median posttransplant follow-up, only reported in eight studies, was 20.1 months (95%CI: 12.3 to 27.9, *I*^*2*^ = 96.4%).

#### Oncological Outcomes – Overall Survival at 1- and 3-Year

**Fig. 6:**
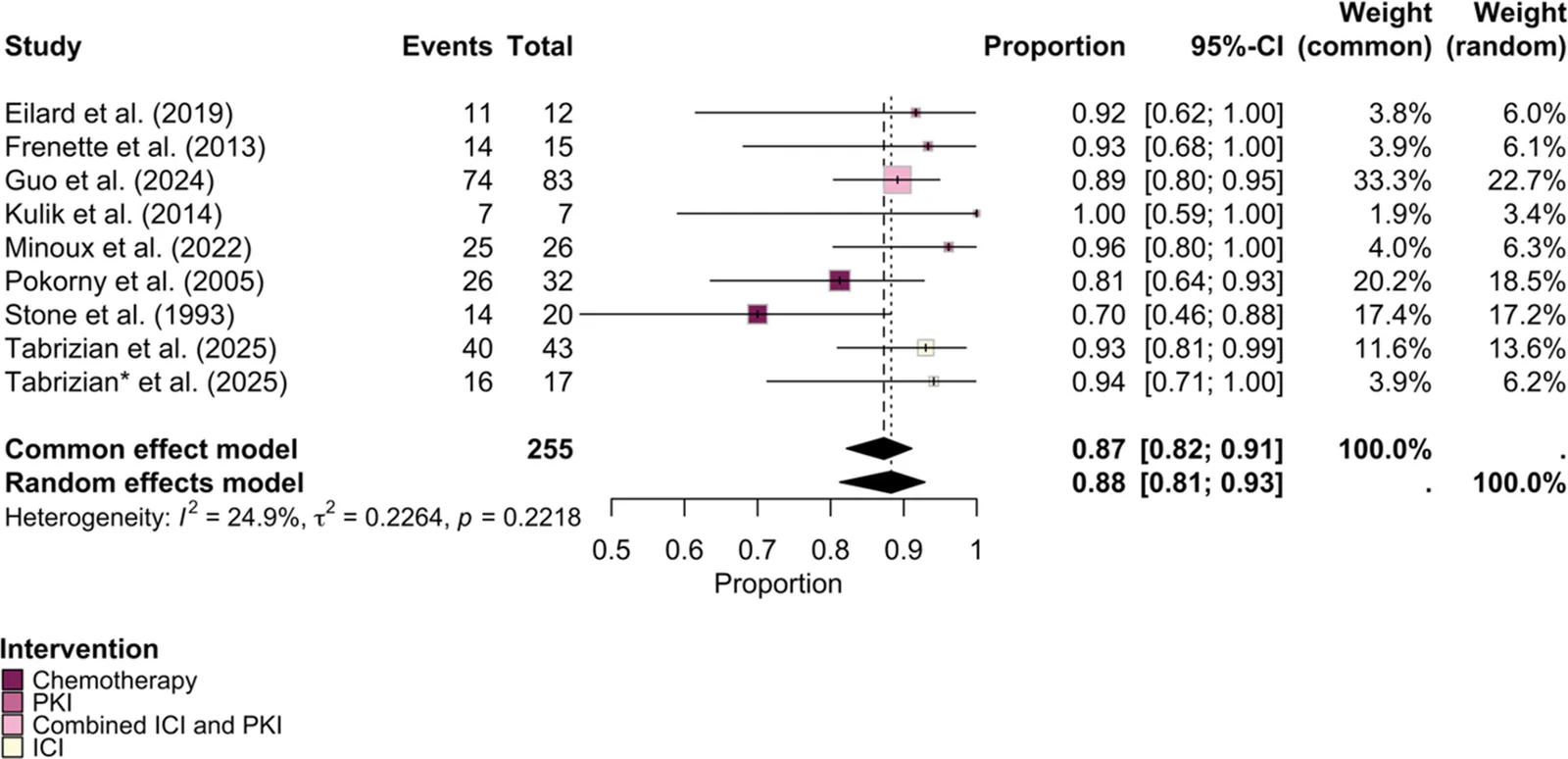
Forest plot of pooled analysis of 1-year overall survival. The pooled OS at 1-year posttransplant is 88% (95%CI: 81% to 93%). Each line displays study details, number of patients, and events. Study-specific cumulative incidence proportions are shown as colored squares with 95%CI. The four different systemic therapy categories (chemotherapy vs. PKI vs. combined PKI-ICI vs. ICI) are represented by distinct colors. Overall pooled cumulative incidence proportions are indicated by black diamonds with their 95%CI. Statistical analysis: Pooled estimates are presented in percentages (%) and were derived using a random-effects model. Heterogeneity was assessed using the *I*^*2*^statistics, *T*^*2*^ and Cochran’s Q *X*^*2*^ test. Significance threshold was set at *p*<0.05. Abbreviations: ICI, Immune checkpoint inhibitor; PKI, Protein kinase inhibitor

**Fig. 7:**
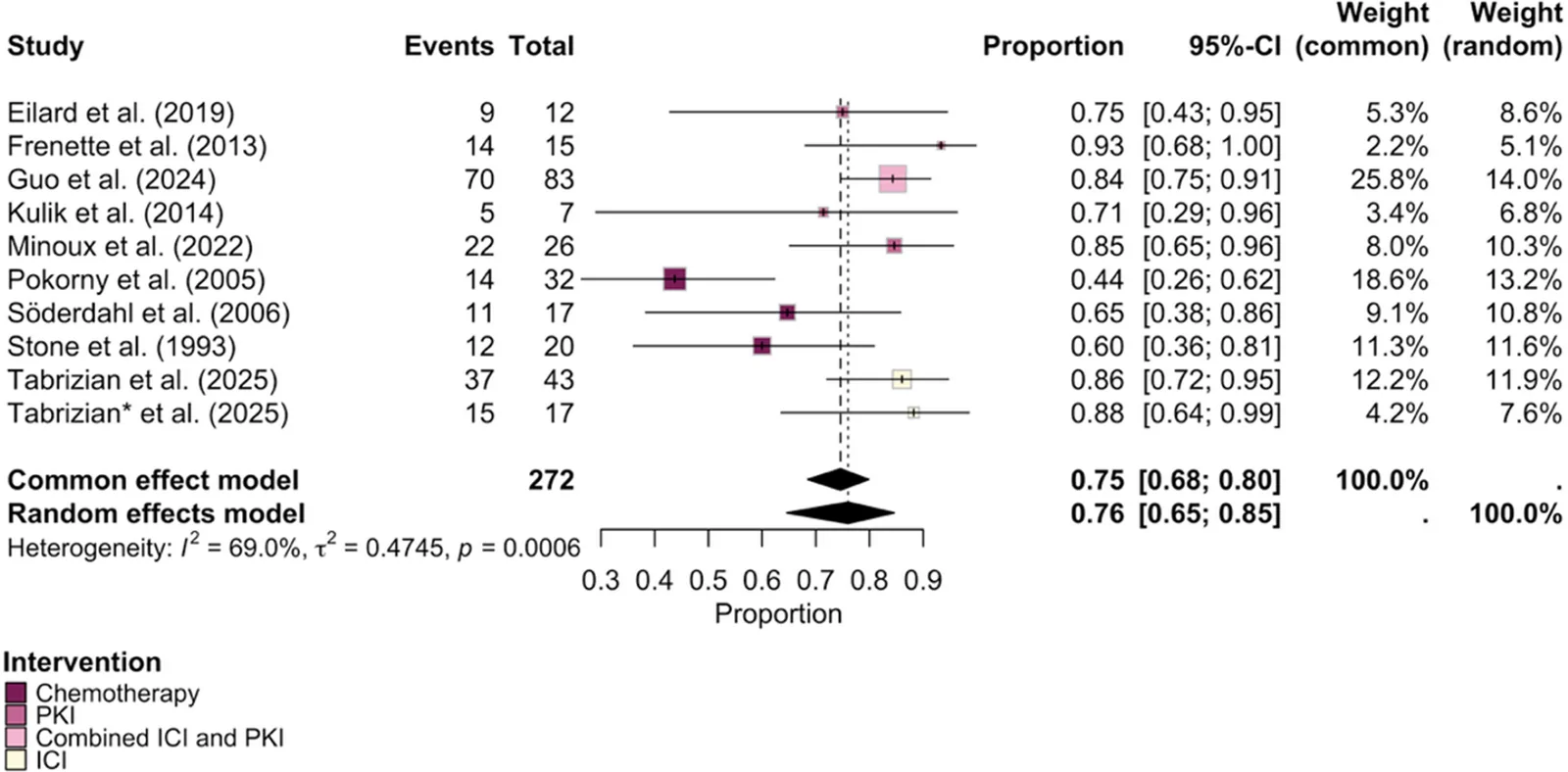
Forest plot of pooled analysis of 3-year overall survival. The pooled OS at 3-year posttransplant is 76% (95%CI: 65% to 85%). Each line displays study details, number of patients, and events. Study-specific cumulative incidence proportions are shown as colored squares with 95%CI. The four different systemic therapy categories (chemotherapy vs. PKI vs. combined PKI-ICI vs. ICI) are represented by distinct colors. Overall pooled cumulative incidence proportions are indicated by black diamonds with their 95%CI. Statistical analysis: Pooled estimates are presented in percentages (%) and were derived using a random-effects model. Heterogeneity was assessed using the *I*^*2*^statistics, *T*^*2*^ and Cochran’s Q *X*^*2*^ test. Significance threshold was set at *p*<0.05. Abbreviations: ICI, Immune checkpoint inhibitor; PKI, Protein kinase inhibitor

Pooled OS decreased throughout the years (see Figs. 6 and 7), with values of 88% (95%CI: 81% to 93%) and 76% (95%CI: 65% to 85%) at 1- and 3-year posttransplant, respectively. In parallel, we observed a trend towards an increasing heterogeneity (*I*^*2*^ of 24.9% and 69.0%). Subgroup pooled analyses (see Online Resources 4–5) showed similar pooled cumulative incidence proportions at 1- and 3-year, except the chemotherapy group (and the oldest studies), with an OS of 77% (95%CI: 64% to 86%) and 54% (95%CI: 42% to 65%) at 1- and 3-year, respectively. Sensitivity analyses excluding that subgroup – at the same time, restricting studies to the most recent cohorts – resulted in comparable pooled cumulative incidence proportions of 92% (95%CI: 87% to 95%, *p* = 0.277) and 84% (95%CI: 78% to 89%, *p* = 0.170); nonetheless, heterogeneity between studies was lower (both *I*^*2*^ = 0.0%).

#### Oncological Outcomes – Recurrence-Free Survival at 1- and 3-Year

RFS also decreased over time, with higher pooled estimates at 1-year (86%, 95%CI: 68% to 94%) than at 3-year (63%, 95%CI: 44% to 79%) (see Online Resources 6–7). Pooled subgroup analyses showed lower cumulative incidence proportions in the chemotherapy group of 69% (95%CI: 56% to 80%) and 57% (95%CI: 43% to 70%), at 1- and 3-year, respectively. This last group was excluded for sensitivity analyses, which resulted in lower heterogeneity (both *I*^*2*^ = 0.0% vs. 50.3% at 1-year and 59.8% at 3-year in the original analyses). Nonetheless, cumulative incidence proportions at 1-year (95%, 95%CI: 84% to 99%) and 3 years (71%, 95%CI: 52% to 84%) were not statistically different from the previous values.

#### Oncological Outcomes – Recurrence and Mortality Proportions

**Fig. 8:**
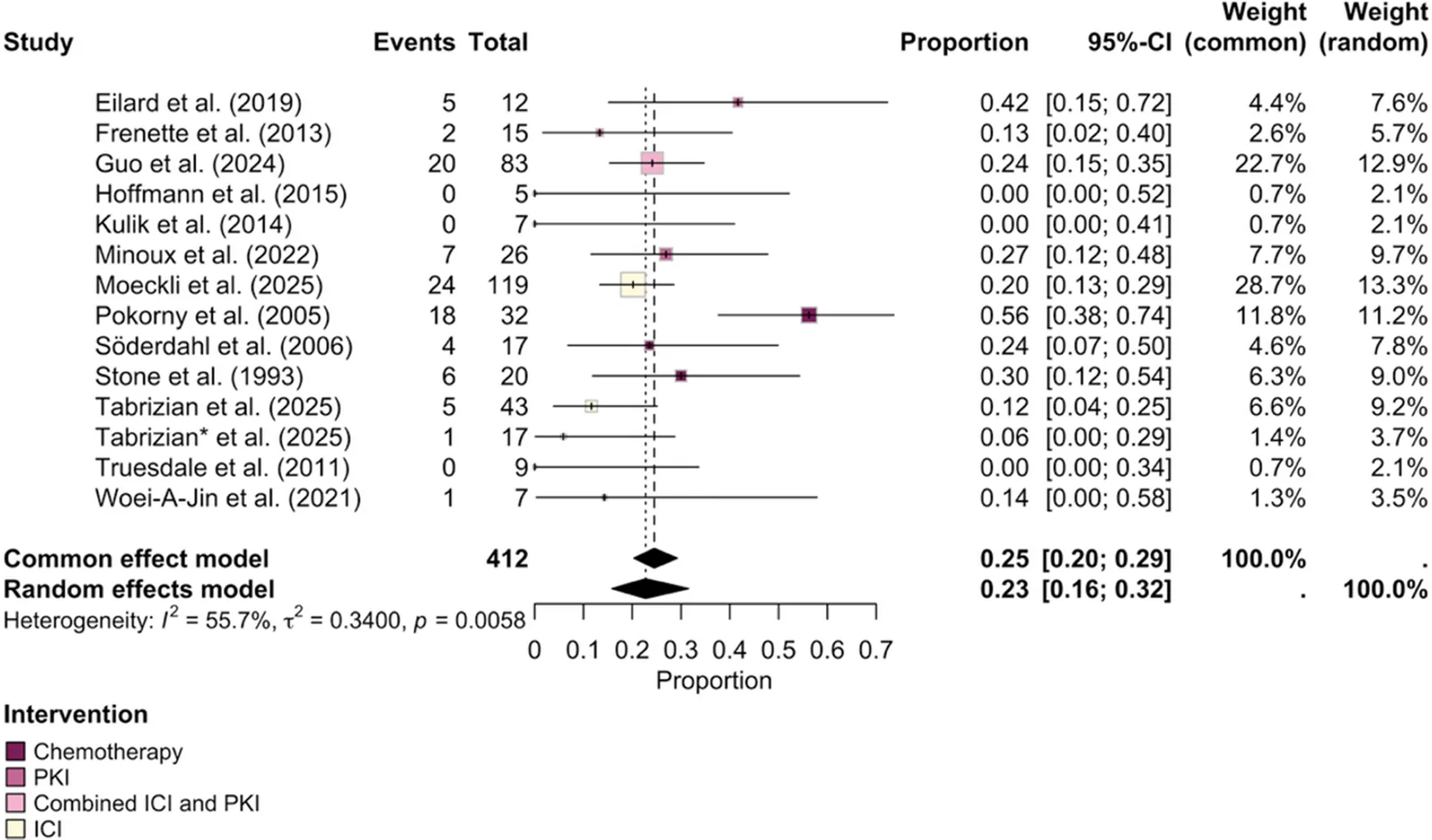
Forest plot of pooled analysis of recurrence proportion. Forest plot displaying a pooled recurrence proportion across studies of 23% (95%CI: 16% to 32%). Statistical analysis: Pooled estimates are presented in percentages (%) and were derived using a random-effects model. Heterogeneity was assessed using the *I*^*2*^statistics, *T*^*2*^ and Cochran’s Q *X*^*2*^ test. Significance threshold was set at *p*<0.05. Abbreviations: ICI, Immune checkpoint inhibitor; PKI, Protein kinase inhibitor

Pooled recurrence proportion was 23% across all studies (95%CI: 16% to 32%), with a high heterogeneity (*I*^*2*^ = 55.7%) (see Fig. 8). Subgroup analysis (see Online Resource 8) showed that patients treated with chemotherapy had a pooled recurrence proportion of 41% (95%CI: 30% to 52%) and high heterogeneity (*I*^*2*^ = 66.6%). Pooled proportions for other subgroups ranged from 17% to 24%, with 95%CI between 11% and 35%.

**Fig. 9:**
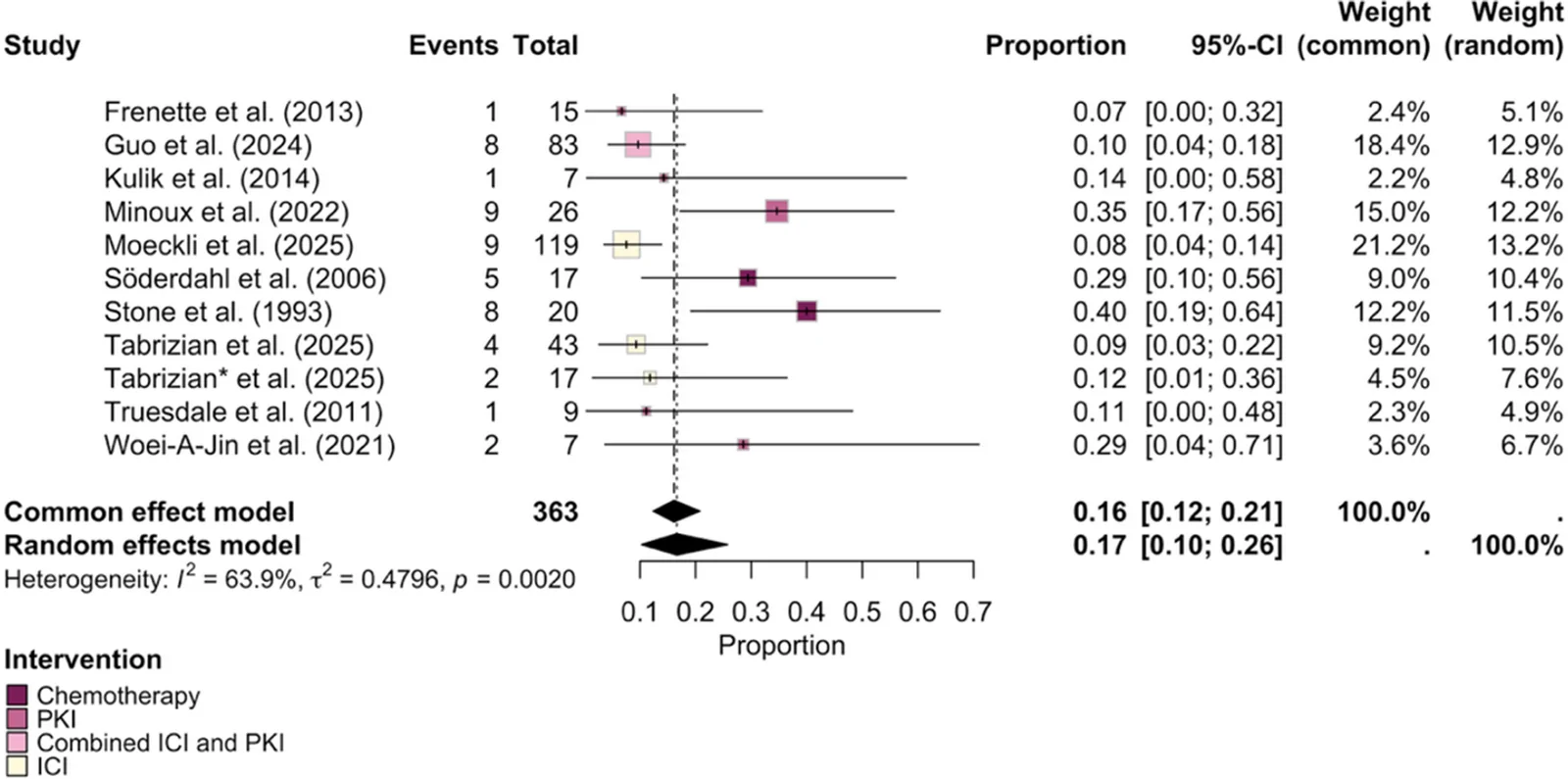
Forest plot of pooled analysis of mortality proportion. Forest plots displaying a pooled mortality proportion of 17% (95%CI: 10% to 26%). Statistical analysis: Pooled estimates are presented in percentages (%) and were derived using a random-effects model. Heterogeneity was assessed using the *I*^*2*^statistics, *T*^*2*^ and Cochran’s Q *X*^*2*^ test. Significance threshold was set at *p*<0.05. Abbreviations: ICI, Immune checkpoint inhibitor; PKI, Protein kinase inhibitor

Pooled mortality proportion was 17% across studies (95%CI: 10% to 26%), with a high heterogeneity (*I*^*2*^ = 63.9%) (see Fig. 9). Subgroup-specific pooled proportions varied. Both ICI and ICI-PKI subgroups displayed low pooled proportions of 8% and 10%, respectively (see Online Resource 9). The PKI group had a pooled mortality proportion of 22% (95%CI: 13% to 34%) and the chemotherapy group had 35% (95%CI: 22% to 52%).

Sensitivity analyses excluding the chemotherapy group resulted in non-statistically different pooled proportions of recurrence (21%, 95%CI: 16% to 25%) and mortality (13%, 95%CI: 8% to 21%).

#### Oncological Outcomes – Median Time-to-Recurrence

Median TTR was available in only four studies, with an overall estimate of 13.5 months (95%CI: 4.7 to 22.4) and a high heterogeneity (*I*^*2*^ = 95.3%). One cohort on PKIs displayed a long TTR (24.9 months), one cohort from the PKI-ICI subgroup had an estimate of 5.5 months, and two cohorts on chemotherapy reported median TTR of 10.5 and 12.5 months.

#### Surgical Outcomes – Acute Cellular Rejection and Graft Loss Proportions

**Fig. 10:**
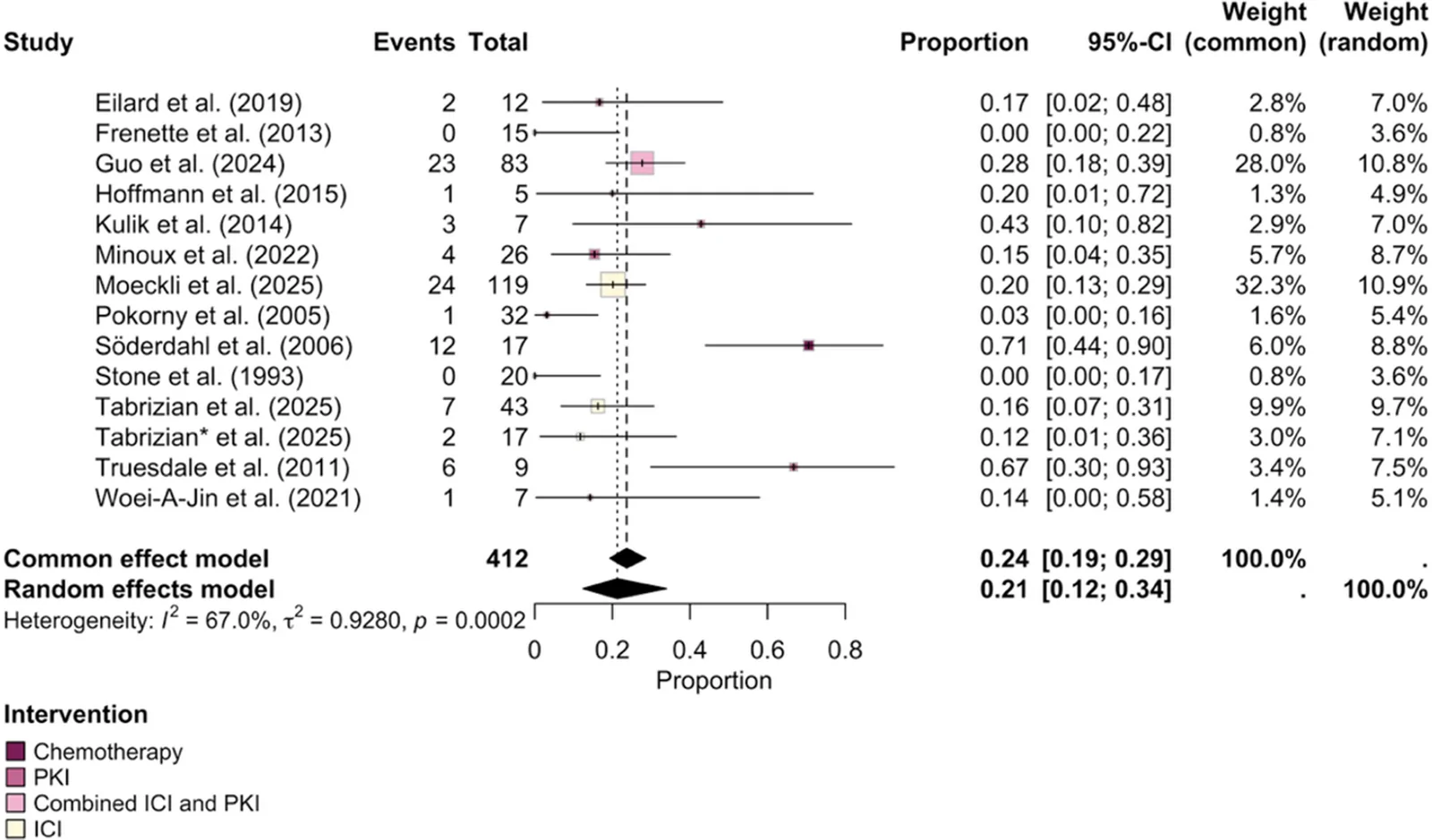
Forest plots of pooled analysis of acute cellular rejection proportion. Forest plots displaying a pooled acute cellular rejection proportion of 21% (95%CI: 12% to 34%). Statistical analysis: Pooled estimates are presented in percentages (%) and were derived using a random-effects model. Heterogeneity was assessed using the *I*^*2*^statistics, *T*^*2*^ and Cochran’s Q *X*^*2*^ test. Significance threshold was set at *p*<0.05. Abbreviations: ICI, Immune checkpoint inhibitor; PKI, Protein kinase inhibitor

ACR was reported in 12 studies involving 86 patients. Most studies (9/12) reported these events in the 30-day posttransplant period; the others reported them over longer time periods, up to 1 year. Diagnostic criteria were explicitly described for 76.7% of patients (*n* = 66); among these, 43 were biopsy-proven, while 23 were based on biological perturbations. The pooled ACR proportion across all patients was 21% (95%CI: 12% to 34%, *I*^*2*^ = 67.0%) (see Fig. 10); the ICI, chemotherapy and PKI groups displayed overall estimates of 18%, 19% and 21%, respectively (see Online Resource 10). The cohort treated with PKI-ICI displayed a proportion of 28% (95%CI: 18% to 39%). Overall and subgroup-specific heterogeneity was high, independent of the study design, the systemic treatment, and the diagnostic criteria (see Online Resource 11, which displays forest plots by diagnostic criteria).

Graft loss was an event with a pooled proportion of 7% across all studies (95%CI: 4% to 11%) and a moderate heterogeneity (*I*^*2*^ = 15.6%) (see Online Resource 12). The chemotherapy group had a pooled estimate of 0%, ICI 4%, PKI-ICI 7% and PKI 5%. Sensitivity analyses yielded similar pooled proportions for rejection and graft loss.

#### Surgical Outcomes – Major Postoperative Complications Rates

Pooled rate of postoperative complications CDC ≥ IIIa was 28% (95%CI: 14% to 48%, *I*^*2*^ = 82.8%) (see Online Resource 13). The PKI group stood out with an overall rate of 47% (95%CI: 36% to 59%); other groups showed pooled estimates ranging from 7% to 15%, with 95%CI between 3% and 38% (see Online Resource 14). Sensitivity analysis excluding the six PKI studies showed a pooled overall proportion of 11% (95%CI: 5% to 23%), not significantly lower than the original analysis (*p* = 0.083).

#### Surgical Outcomes – Median Postoperative Length of Stay

Median postoperative LOS – pooled from 3 studies only – was 16.4 days (95%CI: 11.7 to 21.2, *I*^*2*^ = 41.4%).

## Discussion

### Headlines

This review pooled important survival outcomes, including 1- and 3-year posttransplant OS and RFS, across different HCC treatments. Sensitivity analyses excluding the chemotherapy subgroup led to higher pooled cumulative incidence proportions survival estimates and lower proportions for mortality and recurrence, though not statistically significant. These findings may reflect a temporal bias, as only chemotherapy studies had enrolled patients before 2005. Since then, substantial improvements have been made in diagnostic, prognostic and therapeutic domains. Importantly, this may also reflect the favorable impact of complementary LRT, which were not used in these chemotherapy cohorts. On the other hand, the efficacy of traditional chemotherapy regimens for HCC has been shown to be inferior to that of other systemic regimens, and our sensitivity analyses are congruent, though not reaching statistical significance.

Despite the heterogeneity in neoadjuvant systemic regimens included in our review – particularly chemotherapy – the pooled recurrence proportion we obtained was comparable to the 20.1% reported by Ma et al. in a recent global cohort study including 204 patients with pretransplant use of ICIs [56]. They had reported a median time-to-recurrence of 325 days, shorter than our pooled estimate of 13.5 months. Whilst no conclusion can be drawn from this comparison, the similarity in estimates may support the representativeness of our pooled posttransplant outcomes.

The pooled mortality proportion across studies was high; however, ICI-specific mortality was 8%, which is equal to the estimate reported in Liu et al.’s systematic review of 229 patients receiving ICI before LT [57]. We conducted a scatterplot analysis to investigate a possible association between follow-up duration and mortality (see Online Resource 15), assuming that longer follow-up increases the risk of death due to time-related bias. We used Pearson’s *r* to assess linear correlation, which was not significant (*r* = 0.69, *p* = 0.059). Nonetheless, this underscores the importance of time-to-event or cumulative incidence proportions for future studies of time-dependent variables such as mortality.

An interesting result was the pooled ACR proportion in the ICI monotherapy group. While Rezaee-Zavareh et al.’s systematic review on pre-LT use of ICIs (91 patients) reported a pooled estimate of 26.4% [58], our subgroup analysis resulted in 19% (95%CI: 14% to 25%). Our estimate, closer to Moeckli et al.’s 119-patient cohort (20.2%) [17], but still lower, was probably influenced by both 2025 studies by Tabrizian et al. [52, 53] – reporting values of 16.7% and 11.8% – published after both papers. Compared with the other studies included in our analysis, however, we noted no difference in treatment duration or washout period between the two recent studies. Interestingly, both studies were included by Ma et al., resulting in an even lower rejection rate of 17.5% [56]. 

Ultimately, this review aimed to identify areas for improvement in future research, and the risk of bias assessment underscores methodological issues that compromise the quality of the studies. First, incompleteness in data reporting (e.g., timing of ACR and recurrence, postoperative complications) was an issue that reduced the available data pool and, consequently, the power of our analysis. Second, definitions were inconsistent across studies. For example, ACR diagnoses were either biopsy-proven or based on biological findings, and postoperative complications were either not graded or reported only when major (CDC grade ≥ IIIa), which prevented us from summarizing results using standard classifications such as the CDC or the Comprehensive Complication Index^®^ (CCI^®^) [59]. Third, outcome measurements were not conducted at similar time intervals across studies, either precluding or reducing the number of studies available for the pooled analysis of time-dependent proportions. Finally, when reported, follow-up periods were not comparable, ranging from a median of 8.1 months to 60 months. Standardization is needed for a minimal follow-up period, particularly in transplant oncology, where a 5-year timeframe is appropriate, as used in benchmark studies [60]. 

### Strengths

To our knowledge, this is the first systematic review encompassing the use of systemic therapies for HCC in the neoadjuvant setting before LT.

We adopted a structured and systematic approach during literature search, study screening and selection. We aimed to minimize duplicates; although time-consuming, we thoroughly compared hospitals, sample sizes, studies’ periods and regimens received. Statistical analyses were performed using medians to pool time-dependent variables with non-normal distributions and to account for small study sample sizes. Results of subgroup analyses were presented as objectively and descriptively as possible, to avoid adopting a comparative approach, which would have been inappropriate given our methodology. Finally, heterogeneity was investigated through diverse strategies, such as color-codified forest plots, *I*^*2*^, *T*^*2*^, Cochran’s Q *X*^*2*^ test, subgroup pooled analyses and additional sensitivity analyses. Except for cumulative incidence proportions at 1- and 3-year, most sensitivity analyses excluding the chemotherapy subgroup – thereby restricting analyses to cohorts enrolled since 2005 – resulted in similar pooled estimates, strengthening the robustness of our findings.

### Limitations

Besides the areas of improvement intrinsic to our study pool, which we already detailed, we must acknowledge six limitations. First, we did not restrict our study pool to RCTs, and most studies were single-arm cohorts; therefore, different tools were used for risk-of-bias and quality assessments, limiting comparisons across all studies. Second, these assessments were also done by a single author twice, which may introduce bias despite good intra-rater reliability. Third, screening did not include grey or other unpublished literature and was language-restricted; therefore, publication bias cannot be excluded. Fourth, despite reflecting the real-world target patient population referred to LT, this group is intrinsically heterogeneous in terms of indication (downstaging versus bridging), BCLC stage (A-D), number of treatment cycles, washout periods, and LCT modalities. Although internally valid, our pooled estimates cannot be generalized to highly selected patient populations. Fifth, besides the 26 overlapping cases in the ICI group, one study classified under ICI included eleven patients receiving PKI-ICI combination therapy [17], and the cohort study on PKI-ICI combination comprised four patients treated solely with ICIs [45]. These two misclassifications accounted for 2.7% and 1.0% of the total sample, respectively; therefore, we considered their impact as non-meaningful. Finally, our systematic review was designed to estimate pooled effects and to use subgroups defined by treatments to investigate heterogeneity, not to compare treatment effects directly. Therefore, the results of subgroup pooled analyses are purely descriptive.

## Conclusion and Perspectives

This systematic review pooled posttransplant cumulative incidence proportions in patients with HCC who received systemic therapies prior to LT. These pooled estimates may serve as reference values to support future research on the interpretation of outcomes in transplant-eligible HCC patients, regardless of initial BCLC stage. We also identified areas for improvement in future research to standardize data definitions, measurement, collection, and reporting; increase statistical power; and allow precise estimates, critical for producing clinically actionable evidence. As we did not intend to draw comparisons between neoadjuvant systemic regimens, and the number of published RCTs in this topic may still be limited to conduct high-evidence-level meta-analyses, we await further RCTs. Incorporating such RCTs into a meta-analysis would yield higher-quality evidence on the impact of current therapies – PKIs, ICIs, and their combination – on posttransplant outcomes, including rejection, recurrence, and survival. Ensuring the inferential effect of each treatment on these outcomes and comparing them would lead to improve current clinical practice.

## Supplementary Information

Below is the link to the electronic supplementary material.

Supplementary File 1 (PDF 2.76 MB)

## Data Availability

The datasets generated during and analyzed during the current study are available from the corresponding author on reasonable request.
