# Supplementary material for: Pretransplant Systemic Therapies for Hepatocellular Carcinoma: A Systematic Review of Posttransplant Outcomes
Source: J Gastrointest Cancer. 2026 Feb 14;57(1):45. doi: 10.1007/s12029-026-01399-w (PMC13652768; doi:10.1007/s12029-026-01399-w)
Supplement: Supplementary file 1 [file 12029_2026_1399_MOESM1_ESM.pdf]

Electronic Supplementary Materials

Journal of Gastrointestinal Cancer

Updates in the Management of Primary Liver Cancers

Submission ID: 159fca87-59c9-4597-ba1d-3445b601ada4

Pretransplant Systemic Therapies for Hepatocellular Carcinoma: A Systematic Review of Posttransplant Outcomes

Rodrigues Ribeiro J[1,2], Procter C[1], Toso C[1,3]

[1] University Hospitals of Geneva, Abdominal Surgery Division, Department of Surgery, Geneva, Switzerland

[2] Epidemiology, Biostatistics and Prevention Institute, University of Zurich, Zurich, Switzerland

[3] Faculty of Medicine, University of Geneva, Geneva, Switzerland

Corresponding authors:

Christian Toso
Email address: Christian.toso@hug.ch
ORCID: 0000-0003-1652-4522

Rodrigues Ribeiro Joana
Email address: Joana.rodriguesribeiro@uzh.ch
ORCID: 0009-0001-6629-182X

Table of contents

Electronic Supplementary Materials *3*

ESM 1: PRISMA Checklist 3

ESM 2: Statistical methods for median and interquartile ranges estimation........ 6

ESM 3: Search strategy 7

ESM 4: Subgroup-specific forest plot of pooled analysis of 1-year OS 8

ESM 5: Subgroup-specific forest plot of pooled analysis of 3-year OS 9

ESM 6: Forest plot of pooled analysis of 1-year RFS 10

ESM 7: Forest plot of pooled analysis of 3-year RFS 11

ESM 8: Subgroup-specific forest plot of pooled analysis of recurrence proportions 12

ESM 9: Subgroup-specific forest plot of pooled analysis of mortality proportions 13

ESM 10: Subgroup-specific forest plot of pooled analysis of acute cellular rejection proportion 14

ESM 11: Subgroup-specific forest plot of pooled analysis of acute cellular rejection proportion differentiating diagnostic criteria 15

ESM 12: Forest plot of pooled analysis of graft loss proportion 16

ESM 13: Forest plot of pooled analysis of major postoperative complications rate 17

ESM 14: Subgroup-specific forest plot of pooled analysis of major postoperative complications rates 18

ESM 15: Scatter plot representing mortality proportion over median follow-up 19

References *20*

Electronic Supplementary Materials

ESM 1: PRISMA Checklist

Section and Topic	Item #	Checklist item	Location where item is reported
**TITLE**			
Title	1	Identify the report as a systematic review.	Page 1
**ABSTRACT**			
Abstract	2	See the PRISMA 2020 for Abstracts checklist.	Page 2
**INTRODUCTION**			
Rationale	3	Describe the rationale for the review in the context of existing knowledge.	Pages 3-4
Objectives	4	Provide an explicit statement of the objective(s) or question(s) the review addresses.	Page 4
**METHODS**			
Eligibility criteria	5	Specify the inclusion and exclusion criteria for the review and how studies were grouped for the syntheses.	Pages 5-6
Information sources	6	Specify all databases, registers, websites, organisations, reference lists and other sources searched or consulted to identify studies. Specify the date when each source was last searched or consulted.	Page 6
Search strategy	7	Present the full search strategies for all databases, registers and websites, including any filters and limits used.	ESM 2
Selection process	8	Specify the methods used to decide whether a study met the inclusion criteria of the review, including how many reviewers screened each record and each report retrieved, whether they worked independently, and if applicable, details of automation tools used in the process.	Pages 6-7
Data collection process	9	Specify the methods used to collect data from reports, including how many reviewers collected data from each report, whether they worked independently, any processes for obtaining or confirming data from study investigators, and if applicable, details of automation tools used in the process.	Page 7
Data items	10a	List and define all outcomes for which data were sought. Specify whether all results that were compatible with each outcome domain in each study were sought (e.g. for all measures, time points, analyses), and if not, the methods used to decide which results to collect.	Pages 7, 9
	10b	List and define all other variables for which data were sought (e.g. participant and intervention characteristics, funding sources). Describe any assumptions made about any missing or unclear information.	Page 7
Study risk of bias assessment	11	Specify the methods used to assess risk of bias in the included studies, including details of the tool(s) used, how many reviewers assessed each study and whether they worked independently, and if applicable, details of automation tools used in the process.	Page 8

Section and Topic	Item #	Checklist item	Location where item is reported
Effect measures	12	Specify for each outcome the effect measure(s) (e.g. risk ratio, mean difference) used in the synthesis or presentation of results.	Page 9
Synthesis methods	13a	Describe the processes used to decide which studies were eligible for each synthesis (e.g. tabulating the study intervention characteristics and comparing against the planned groups for each synthesis (item #5)).	Page 9
	13b	Describe any methods required to prepare the data for presentation or synthesis, such as handling of missing summary statistics, or data conversions.	Pages 6-8
	13c	Describe any methods used to tabulate or visually display results of individual studies and syntheses.	Pages 8-9
	13d	Describe any methods used to synthesize results and provide a rationale for the choice(s). If meta-analysis was performed, describe the model(s), method(s) to identify the presence and extent of statistical heterogeneity, and software package(s) used.	Page 9
	13e	Describe any methods used to explore possible causes of heterogeneity among study results (e.g. subgroup analysis, meta-regression).	Page 9
	13f	Describe any sensitivity analyses conducted to assess robustness of the synthesized results.	Page 9
Reporting bias assessment	14	Describe any methods used to assess risk of bias due to missing results in a synthesis (arising from reporting biases).	Page 9
Certainty assessment	15	Describe any methods used to assess certainty (or confidence) in the body of evidence for an outcome.	Page 9
**RESULTS**			
Study selection	16a	Describe the results of the search and selection process, from the number of records identified in the search to the number of studies included in the review, ideally using a flow diagram.	Page 10
	16b	Cite studies that might appear to meet the inclusion criteria, but which were excluded, and explain why they were excluded.	Page 10
Study characteristics	17	Cite each included study and present its characteristics.	Page 10
Risk of bias in studies	18	Present assessments of risk of bias for each included study.	Page 11 + Figures
Results of individual studies	19	For all outcomes, present, for each study: (a) summary statistics for each group (where appropriate) and (b) an effect estimate and its precision (e.g. confidence/credible interval), ideally using structured tables or plots.	Pages 11-13 + Figures + ESM 3-4
Results of syntheses	20a	For each synthesis, briefly summarise the characteristics and risk of bias among contributing studies.	Figures
	20b	Present results of all statistical syntheses conducted. If meta-analysis was done, present for each the summary estimate and its precision (e.g. confidence/credible interval) and measures of statistical heterogeneity. If comparing groups, describe the direction of the effect.	Pages 11-13, Figures + ESM 3-4
	20c	Present results of all investigations of possible causes of heterogeneity among study results.	Pages 11-13 + ESM

Section and Topic	Item #	Checklist item	Location where item is reported
	20d	Present results of all sensitivity analyses conducted to assess the robustness of the synthesized results.	Pages 11-13 + ESM
Reporting biases	21	Present assessments of risk of bias due to missing results (arising from reporting biases) for each synthesis assessed.	Page 13 + Figure
	22	Present assessments of certainty (or confidence) in the body of evidence for each outcome assessed.	Page 13
**DISCUSSION**			
Discussion	23a	Provide a general interpretation of the results in the context of other evidence.	Page 14
	23b	Discuss any limitations of the evidence included in the review.	Page 15
	23c	Discuss any limitations of the review processes used.	Page 15
	23d	Discuss implications of the results for practice, policy, and future research.	Page 15
**OTHER INFORMATION**			
Registration and protocol	24a	Provide registration information for the review, including register name and registration number, or state that the review was not registered.	Page 5
	24b	Indicate where the review protocol can be accessed, or state that a protocol was not prepared.	Page 5
	24c	Describe and explain any amendments to information provided at registration or in the protocol.	Page 5, 7-8
Support	25	Describe sources of financial or non-financial support for the review, and the role of the funders or sponsors in the review.	Page 21
Competing interests	26	Declare any competing interests of review authors.	Page 21
Availability of data, code and other materials	27	Report which of the following are publicly available and where they can be found: template data collection forms; data extracted from included studies; data used for all analyses; analytic code; any other materials used in the review.	Page 21

ESM 1 PRISMA checklist from 2020 statement[1]. This work is licensed under CC BY 4.0. To view a copy of this license, visit https://creativecommons.org/licenses/by/4.0/

ESM 2: Statistical methods for median and interquartile ranges estimation

We chose to use median and interquartile ranges (IQRs). When median and ranges (minimum (min) and maximum (max)) were reported for a variable, we assumed a normal distribution of the variable with IQR ≈ 1.349 • standard deviation (SD), used standard normal cumulative distribution function ϕ, and estimated SD with Wan et al.'s method, n being equal to sample size[2]. In case IQR was found to be equal or superior to the median, the inferior interquartile value was set to 0.

$$SD = \frac{(\max - \min)}{2 \cdot \phi^{-1}(\frac{n - 0.375}{n + 0.25})}$$

From which we deducted:

$$IQR \approx 0.349 \bullet \frac{(\max - \min)}{2 \cdot \phi^{-1}(\frac{n - 0.375}{n + 0.25})}$$

When mean and ranges were reported for a variable, we estimated median with Hozo et al.'s method[3].

$$Mean \approx \frac{\min + 2 \cdot median + max}{4}$$

From which we deducted:

$$Median \approx \frac{(4 \cdot Mean) - (\min + \max)}{2}$$

ESM 3: Search strategy

PubMed:

(("hepatocellular carcinoma"[tiab] OR hcc[tiab] OR "primary liver cancer"[tiab]) AND ("liver transplant*"[tiab]))

AND

(("bridging"[tiab] OR "downstaging"[tiab] OR "neoadjuvant"[tiab] OR "before LT"[tiab] OR "before OLT"[tiab] OR "before transplant*"[tiab] OR "before liver transplant*"[tiab] OR "prior to LT"[tiab] OR "prior to liver transplant*"[tiab] OR "prior to OLT"[tiab] OR "pre-transplant*"[tiab] OR "pretransplant*"[tiab] OR "prior to transplant*"[tiab]) OR "neoadjuvant therapy"[MeSH Terms])

AND

(("immunotherapy"[tiab] OR "immune checkpoint inhibitors"[tiab] OR "targeted therapy"[tiab] OR "tki"[tiab] OR "chemotherapy"[tiab] OR "lenvatinib"[tiab] OR "sorafenib"[tiab]) OR "immunotherapy"[MeSH Terms] OR "immune checkpoint inhibitors"[MeSH Terms] OR "molecular targeted therapy"[MeSH Terms] OR "protein kinase inhibitors"[MeSH Terms])

Filters applied: Adaptive Clinical Trial, Case Reports, Classical Article, Clinical Study, Clinical Trial, Clinical Trial, Phase I, Clinical Trial, Phase II, Clinical Trial, Phase III, Clinical Trial, Phase IV, Clinical Trial Protocol, Comparative Study, Controlled Clinical Trial, Corrected and Republished Article, Equivalence Trial, Observational Study, Randomized Controlled Trial, Twin Study, Validation Study

EMBASE (Elsevier):

((('hepatocellular carcinoma' OR hcc OR 'primary liver cancer') AND 'liver transplant*'):ti,ab AND ((bridging OR downstaging OR neoadjuvant OR 'before LT' OR 'before OLT' OR 'before transplant*' OR 'before liver transplant*' OR 'prior to LT' OR 'prior to liver transplant*' OR 'prior to OLT' OR pre-transplant* OR 'prior to transplant*' OR pretransplant*):ti,ab OR 'neoadjuvant therapy'/exp) AND ((immunotherapy OR sorafenib OR lenvatinib OR 'immune checkpoint inhibitors' OR 'targeted therapy' OR tki OR chemotherapy):ti,ab OR ‘immunotherapy’/exp OR 'immune checkpoint inhibitors'/exp OR 'molecular targeted therapy'/exp OR 'protein kinase inhibitors'/exp)) AND ('article'/it OR 'article in press'/it OR 'conference paper'/it OR 'data papers'/it OR 'preprint'/it OR 'clinical trial'/it)

MEDLINE (Ovid):

(("hepatocellular carcinoma" or hcc or "primary liver cancer").ti,ab. and ("liver transplant*").ti,ab. and (bridging or downstaging or neoadjuvant or "before LT" or "before OLT" or "before transplant*" or "before liver transplant*" or "prior to LT" or "prior to liver transplant*" or "prior to OLT" or pre-transplant* or "prior to transplant*" or pretransplant*).ti,ab. or exp Neoadjuvant Therapy/) and (immunotherapy or "immune checkpoint inhibitors" or "targeted therapy" or tki or lenvatinib or sorafenib or chemotherapy).ti,ab. or exp Immunotherapy/ or exp Immune Checkpoint Inhibitors/ or exp Molecular Targeted Therapy/ or exp Protein Kinase Inhibitors/) not (exp animals/ not humans/) not (review.pt. or review.ti.)

ESM 4: Subgroup-specific forest plot of pooled analysis of 1-year OS

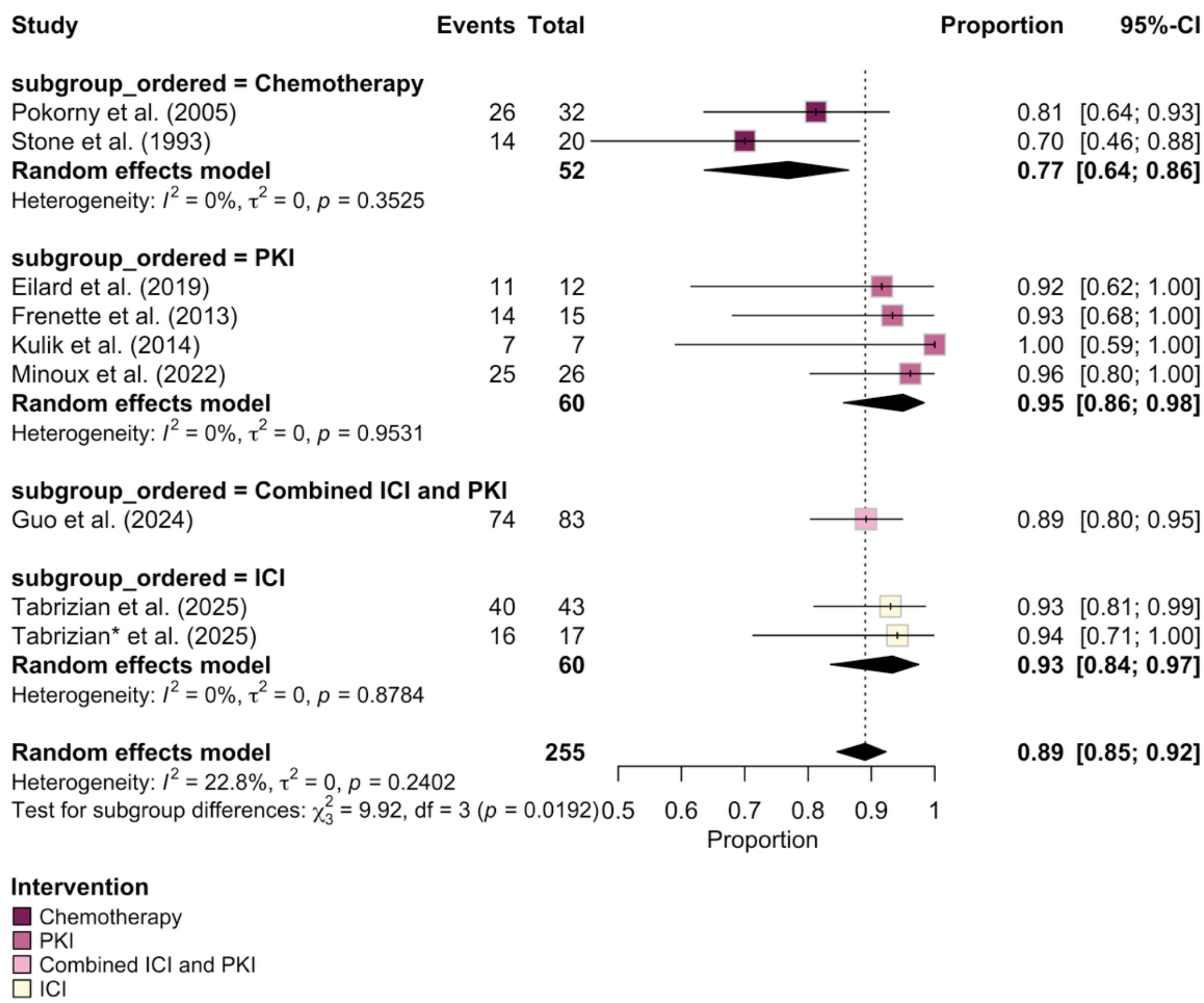

ESM 4 Subgroup-specific forest plot of pooled 1-y OS. It illustrates the pooled estimates within each subgroup, previously defined by the systemic therapy given prior to liver transplant. Statistical analysis: Pooled estimates were derived using a fixed-effect model; any comparison across subgroups is statistically unsound and must not be made. Heterogeneity was assessed using the I^2 statistics, T^2 and Cochran's Q X^2 test. Significance threshold was set at p<0.05.

ESM 5: Subgroup-specific forest plot of pooled analysis of 3-year OS

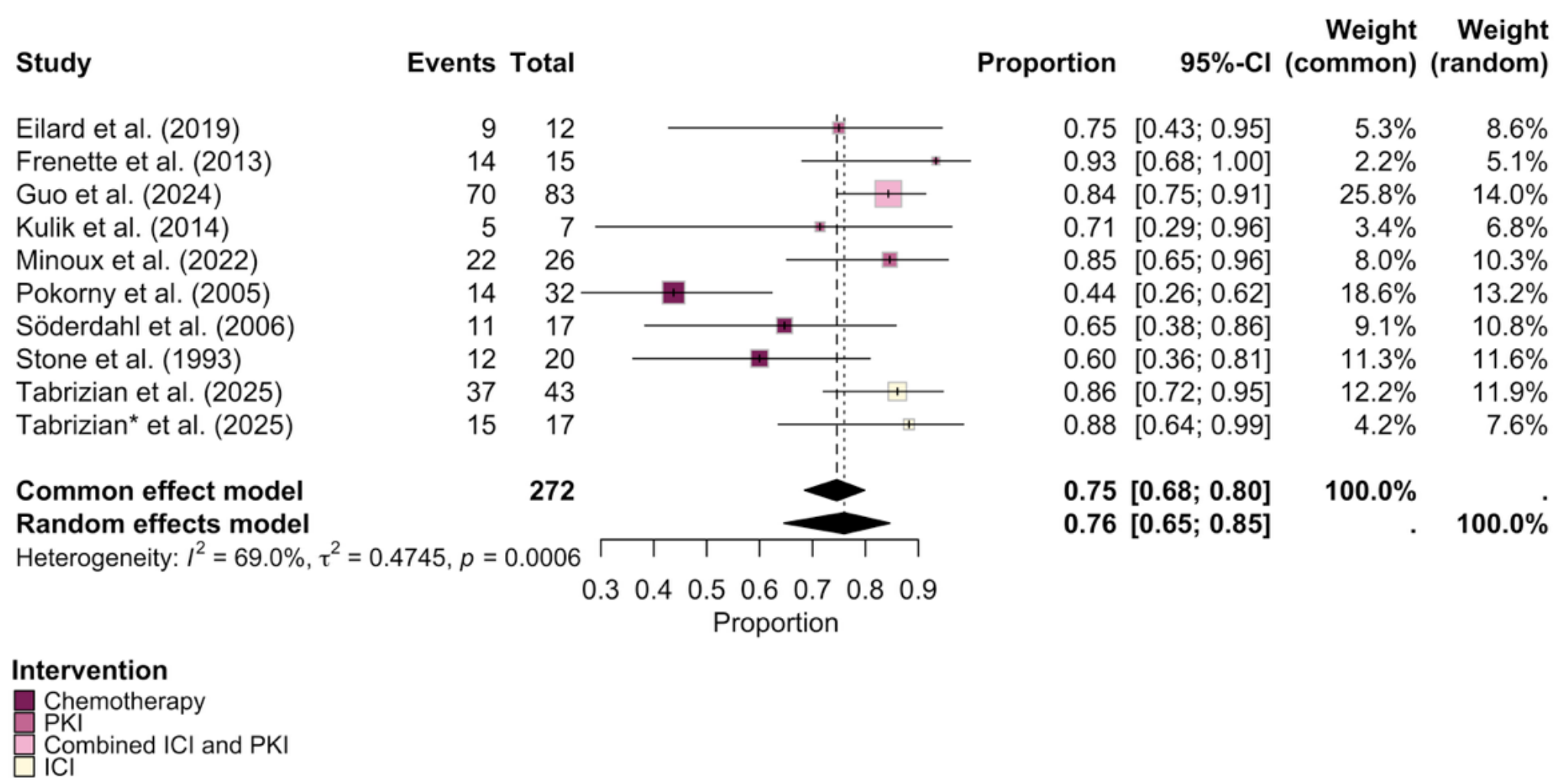

ESM 5 Subgroup-specific forest plot of pooled 3-y OS. It illustrates the pooled estimates within each subgroup, previously defined by the systemic therapy given prior to liver transplant. Statistical analysis: Pooled estimates were derived using a fixed-effect model; any comparison across subgroups is statistically unsound and must not be made. Heterogeneity was assessed using the I^2 statistics, T^2 and Cochran's Q X^2 test. Significance threshold was set at p<0.05.

ESM 6: Forest plot of pooled analysis of 1-year RFS

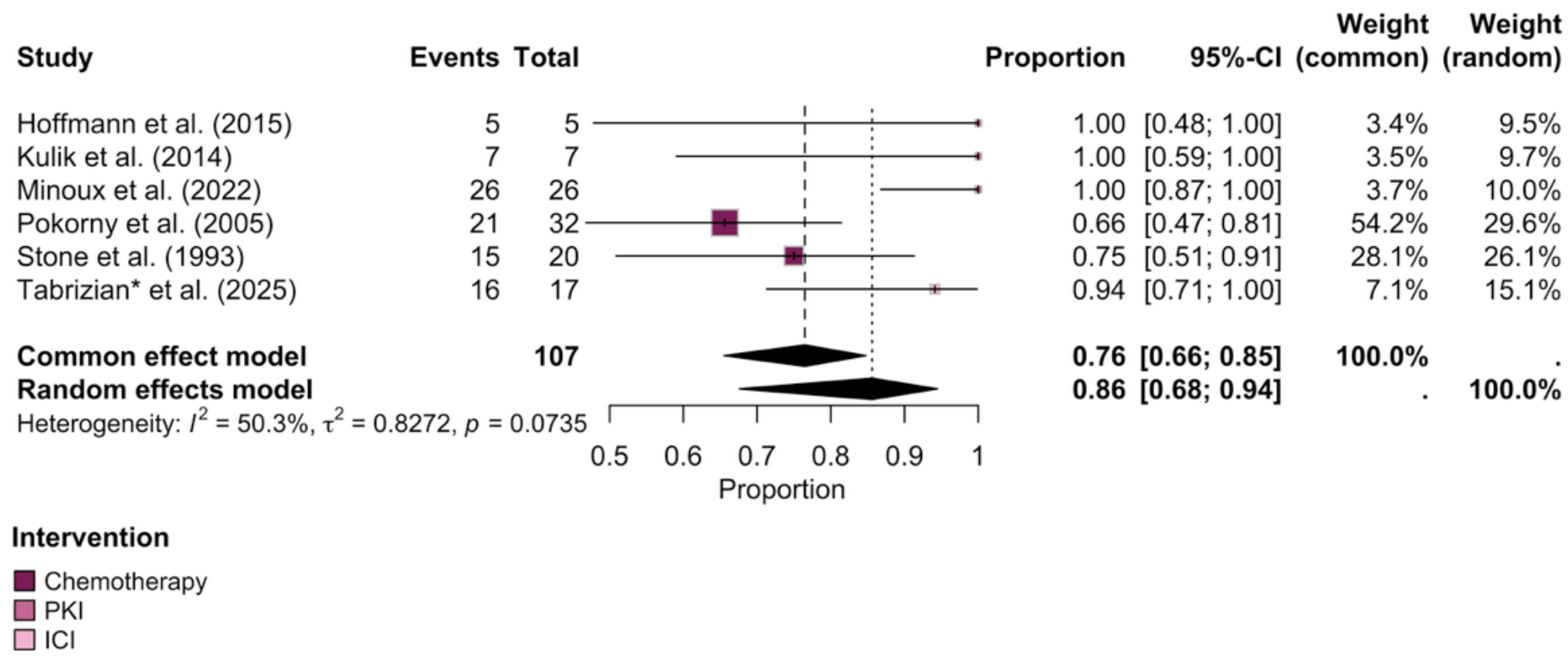

ESM 6 Forest plot of pooled 1-y RFS. It illustrates the pooled cumulative incidence proportion across studies. Statistical analysis: Pooled estimates are presented in percentages (%) and were derived using a random-effects model. Heterogeneity was assessed using the I^2 statistics, T^2 and Cochran's Q X^2 test. Significance threshold was set at p<0.05.

ESM 7: Forest plot of pooled analysis of 3-year RFS

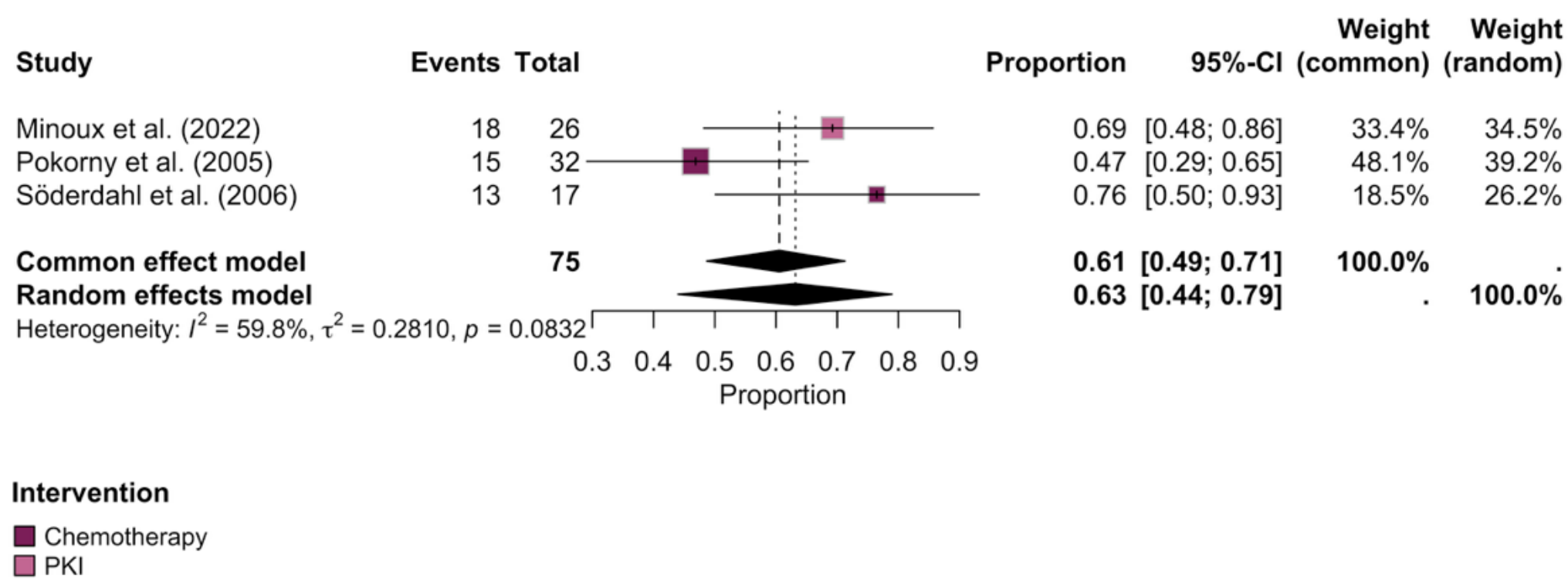

ESM 7 Forest plot of pooled 3-y RFS. It illustrates the pooled cumulative incidence proportion across studies. Statistical analysis: Pooled estimates are presented in percentages (%) and were derived using a random-effects model. Heterogeneity was assessed using the I^2 statistics, T^2 and Cochran's Q X^2 test. Significance threshold was set at $p<0.05$.

ESM 8: Subgroup-specific forest plot of pooled analysis of recurrence proportions

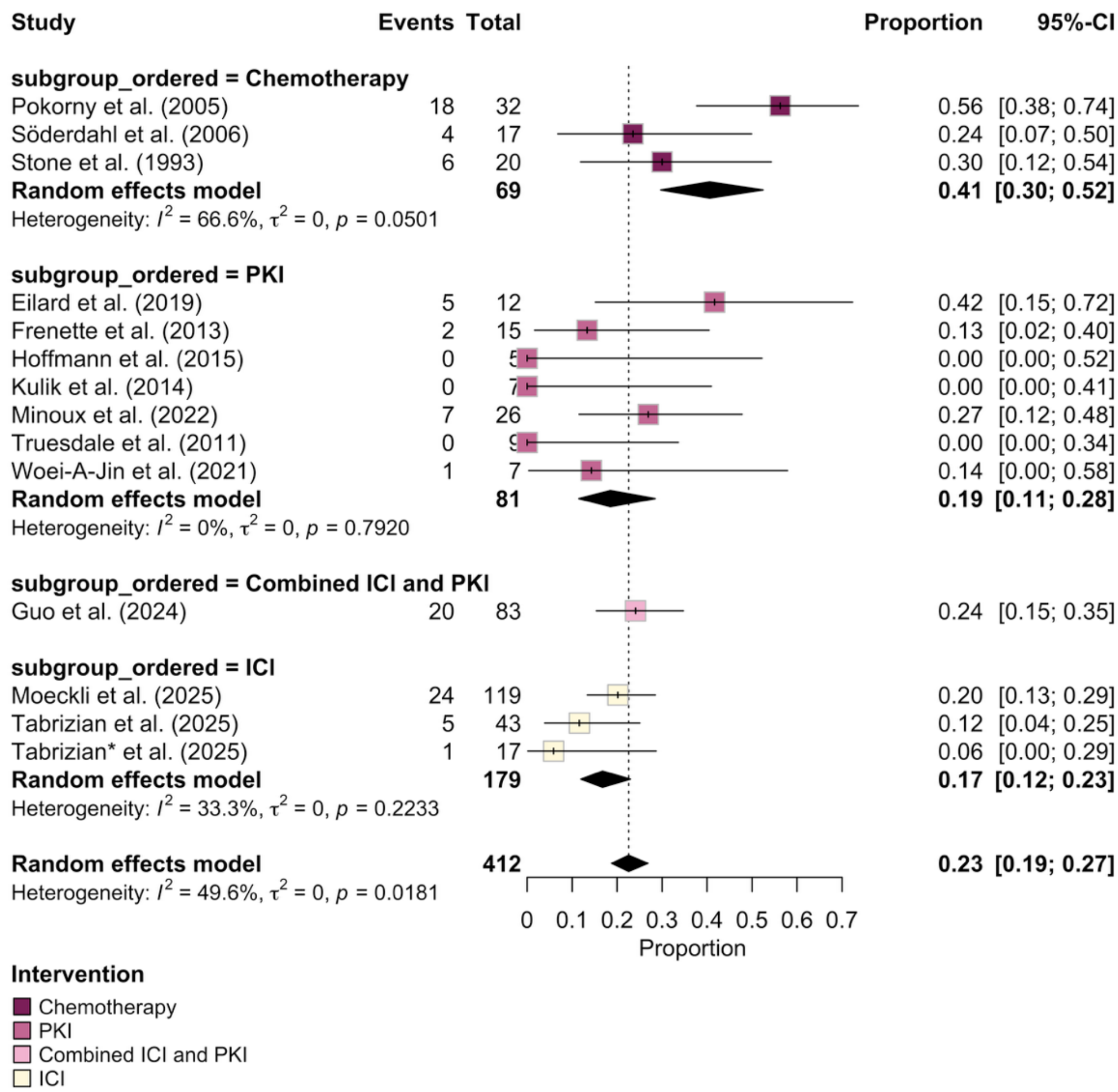

ESM 8 Subgroup-specific forest plot of pooled recurrence proportions. It illustrates the pooled estimates within each subgroup, previously defined by the systemic therapy given prior to liver transplant. Statistical analysis: Pooled estimates were derived using a fixed-effect model; any comparison across subgroups is statistically unsound and must not be made. Heterogeneity was assessed using the I^2 statistics, T^2 and Cochran's Q X^2 test. Significance threshold was set at p<0.05.

ESM 9: Subgroup-specific forest plot of pooled analysis of mortality proportions

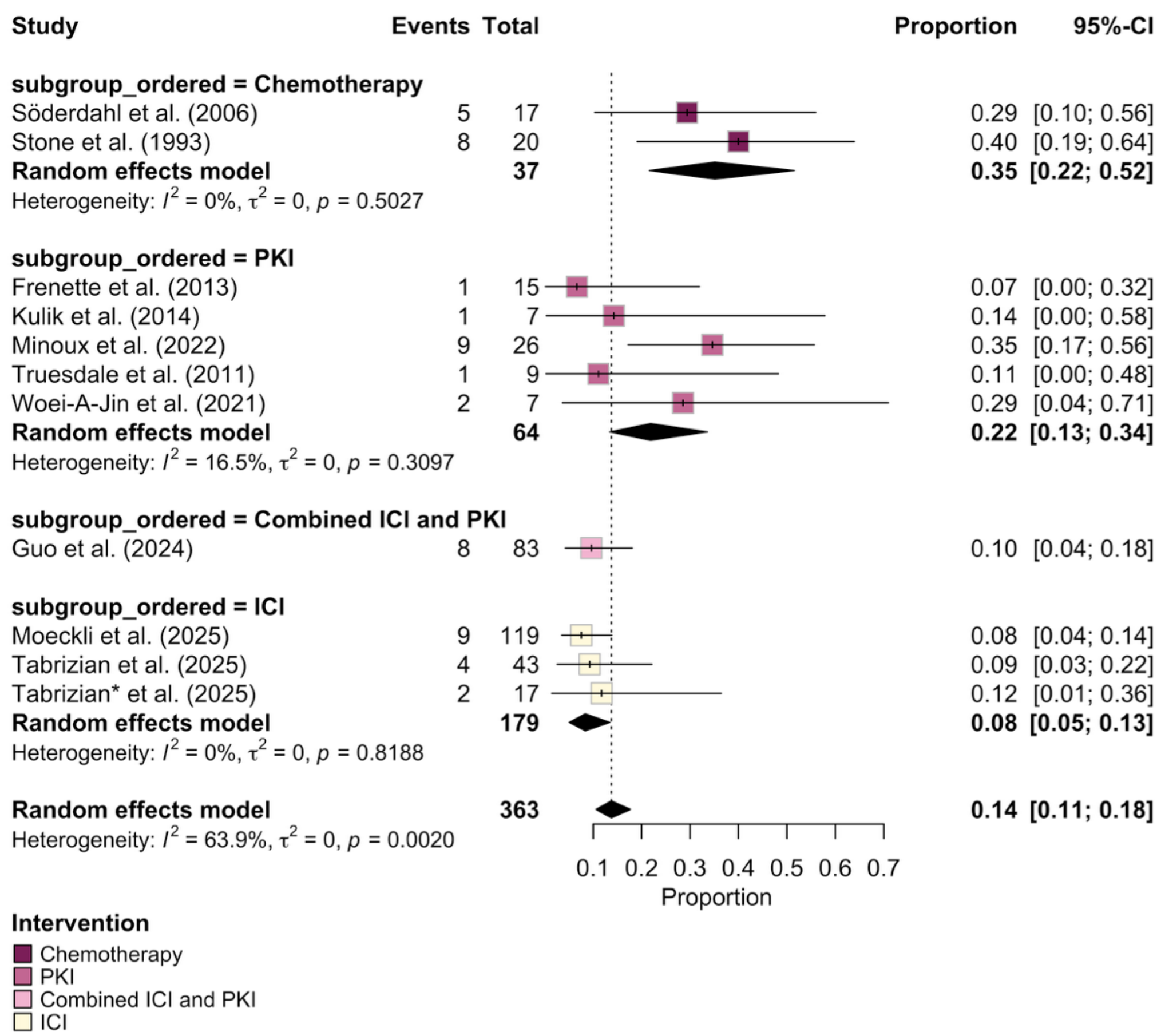

ESM 9 Subgroup-specific forest plot of pooled mortality proportions. It illustrates the pooled estimates within each subgroup, previously defined by the systemic therapy given prior to liver transplant. Statistical analysis: Pooled estimates were derived using a fixed-effect model; any comparison across subgroups is statistically unsound and must not be made. Heterogeneity was assessed using the I^2 statistics, T^2 and Cochran's Q X^2 test. Significance threshold was set at p<0.05.

ESM 10: Subgroup-specific forest plot of pooled analysis of acute cellular rejection proportion

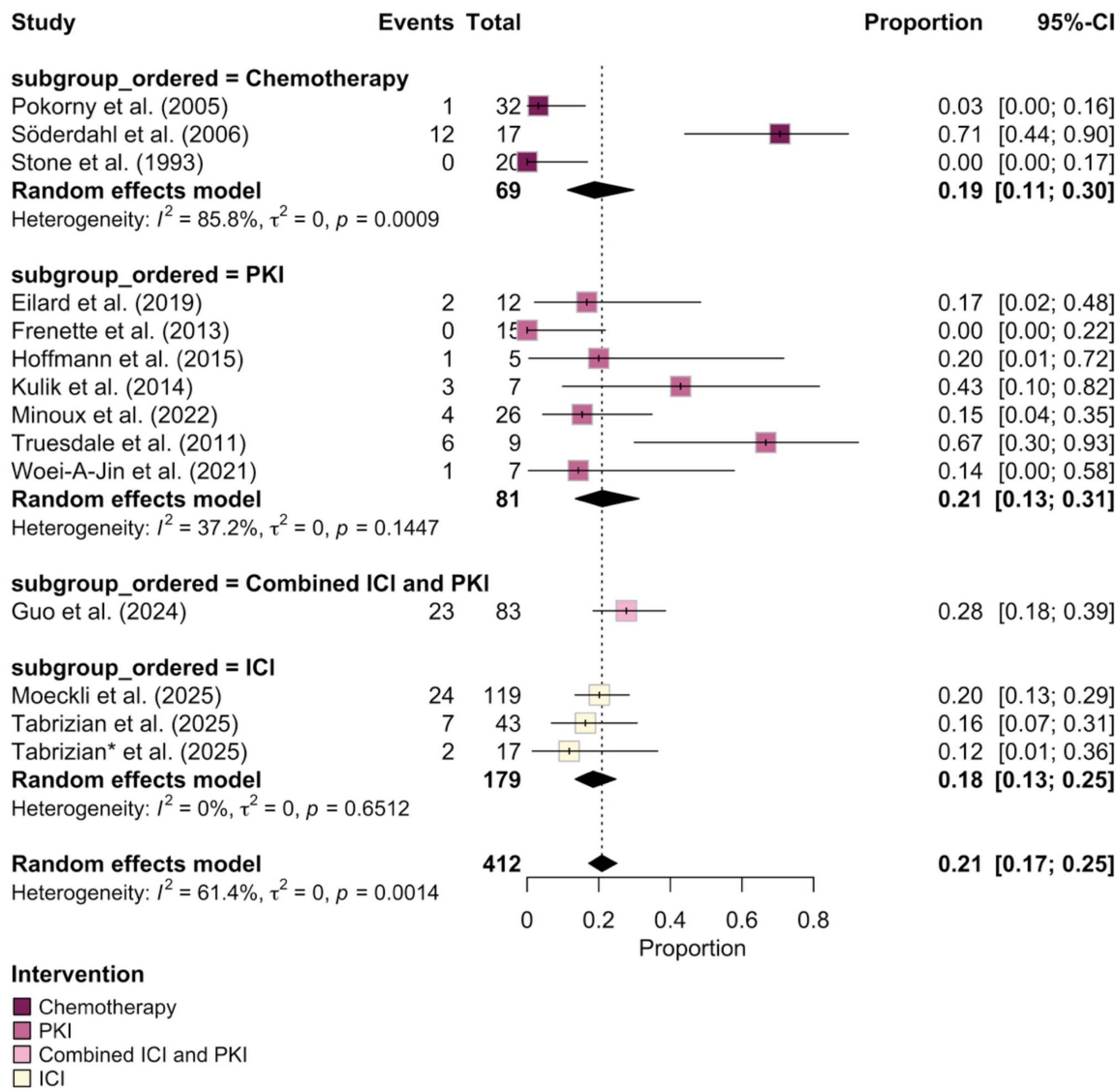

ESM 10 Subgroup-specific forest plot of pooled acute cellular rejection proportions. It illustrates the pooled estimates within each subgroup, previously defined by the systemic therapy given prior to liver transplant. Statistical analysis: Pooled estimates were derived using a fixed-effect model; any comparison across subgroups is statistically unsound and must not be made. Heterogeneity was assessed using the I^2 statistics, T^2 and Cochran's Q X^2 test. Significance threshold was set at *p*<0.05.

ESM 11: Subgroup-specific forest plot of pooled analysis of acute cellular rejection proportion differentiating diagnostic criteria

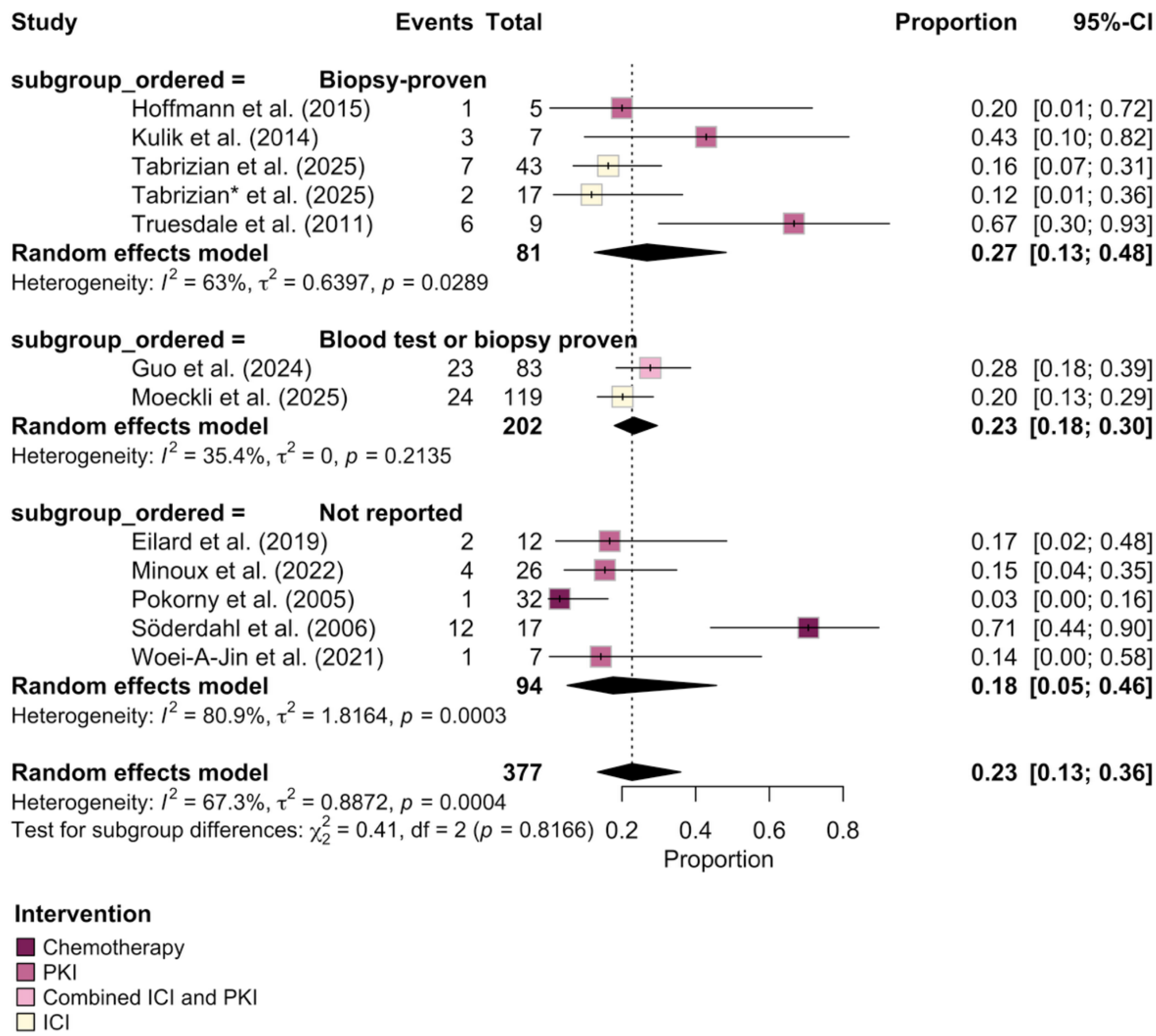

ESM 11 Subgroup-specific forest plot of pooled acute rejection proportions according to diagnostic criteria. It illustrates the pooled estimates within each subgroup, defined by the diagnostic criteria – either biopsy-proven, bimodal, or not reported. Statistical analysis: Pooled estimates were derived using a fixed-effect model; any comparison across subgroups is statistically unsound and must not be made. Heterogeneity was assessed using the I^2 statistics, T^2 and Cochran's Q X^2 test. Significance threshold was set at p<0.05.

ESM 12: Forest plot of pooled analysis of graft loss proportion

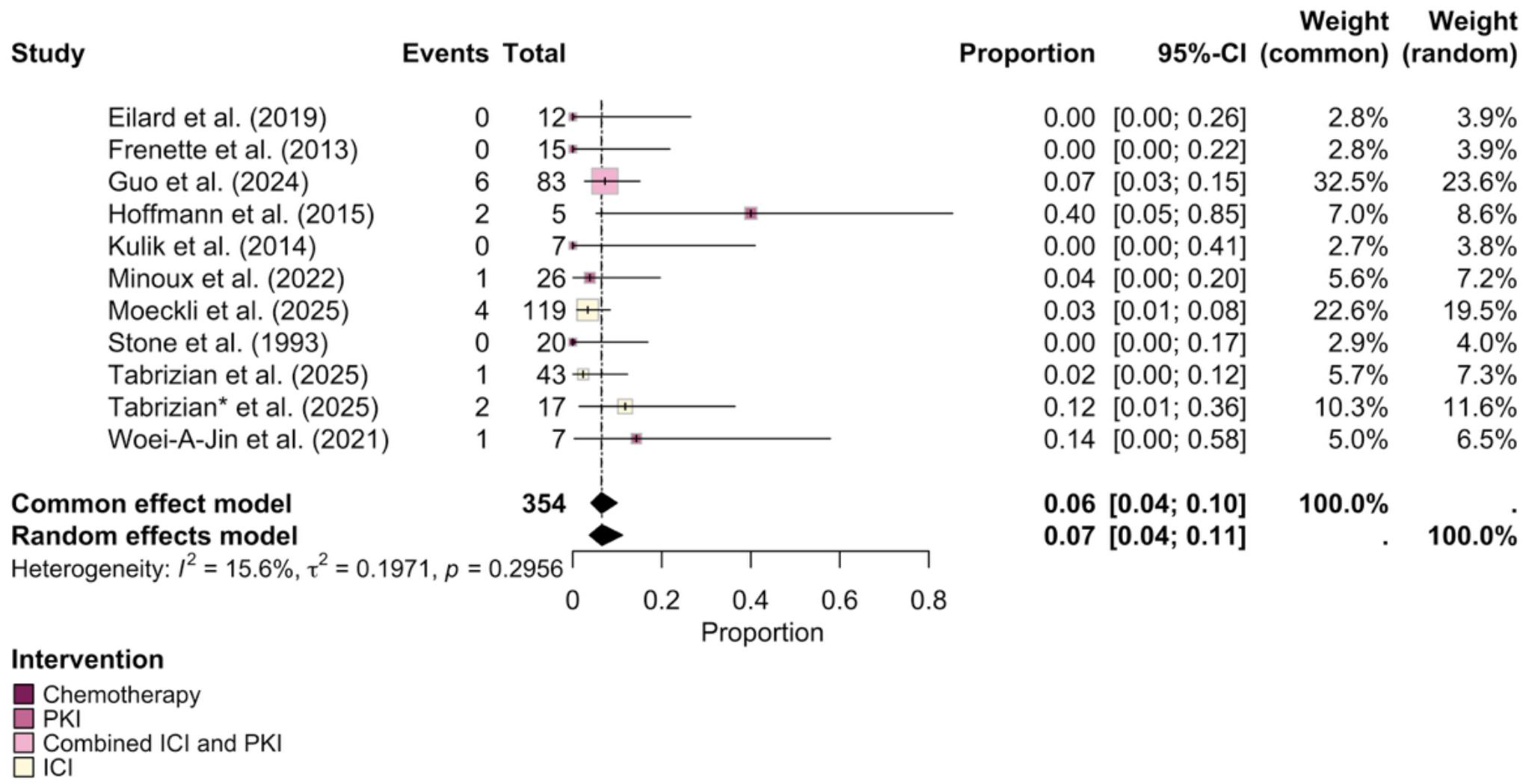

ESM 12 Forest plot of pooled graft loss proportion. It illustrates the pooled cumulative incidence proportion across studies. Statistical analysis: Pooled estimates are presented in percentages (%) and were derived using a random-effects model. Heterogeneity was assessed using the I^2 statistics, T^2 and Cochran's Q X^2 test. Significance threshold was set at p<0.05.

ESM 13: Forest plot of pooled analysis of major postoperative complications rate

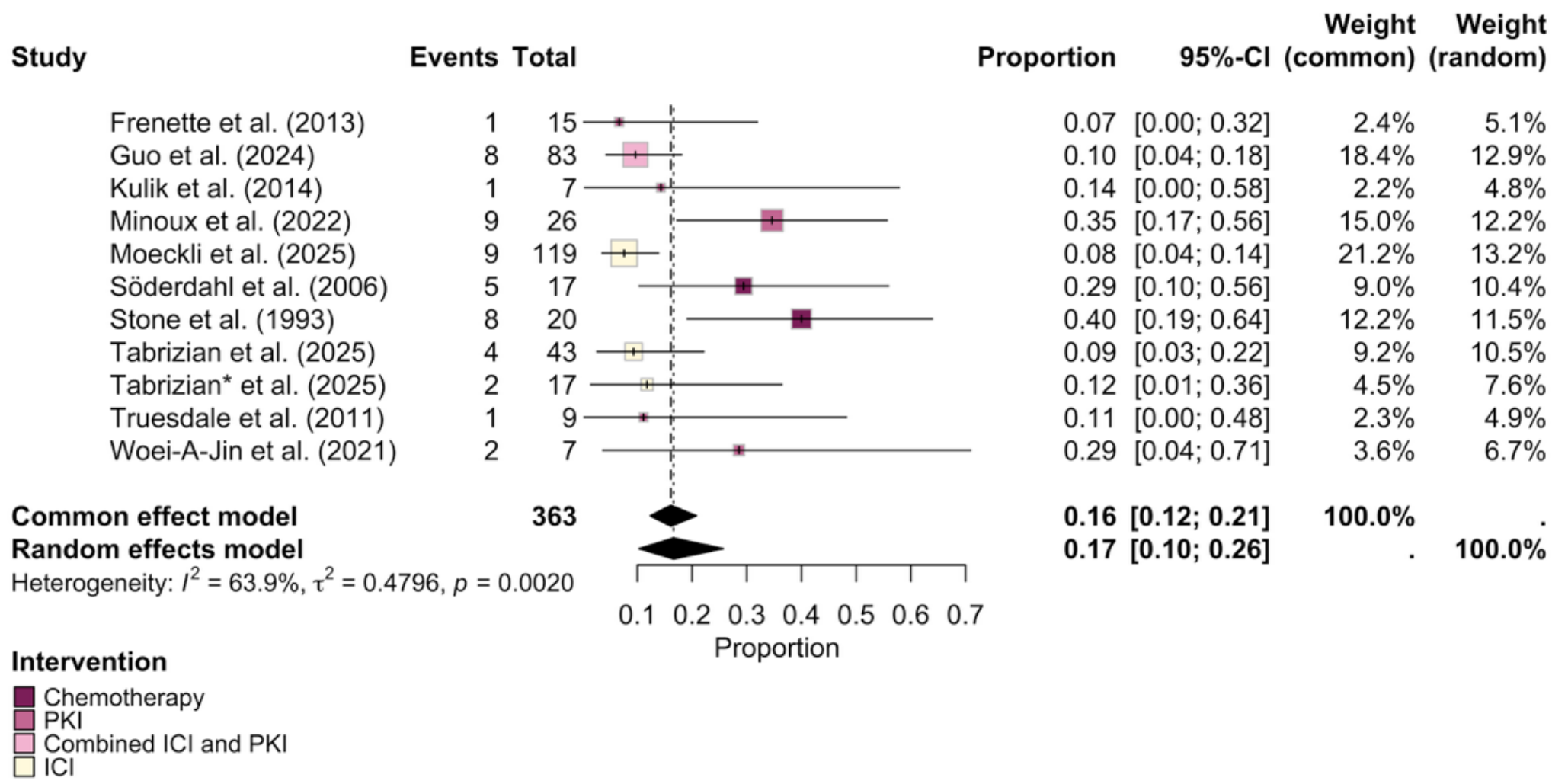

ESM 13 Forest plot of pooled major postoperative complications rate. All forest plots illustrate the pooled estimates across studies. Statistical analysis: Pooled estimates are presented in percentages (%) and were derived using a random-effects model. Heterogeneity was assessed using the I^2 statistics, T^2 and Cochran's Q X^2 test. Significance threshold was set at p<0.05.

ESM 14: Subgroup-specific forest plot of pooled analysis of major postoperative complications rates

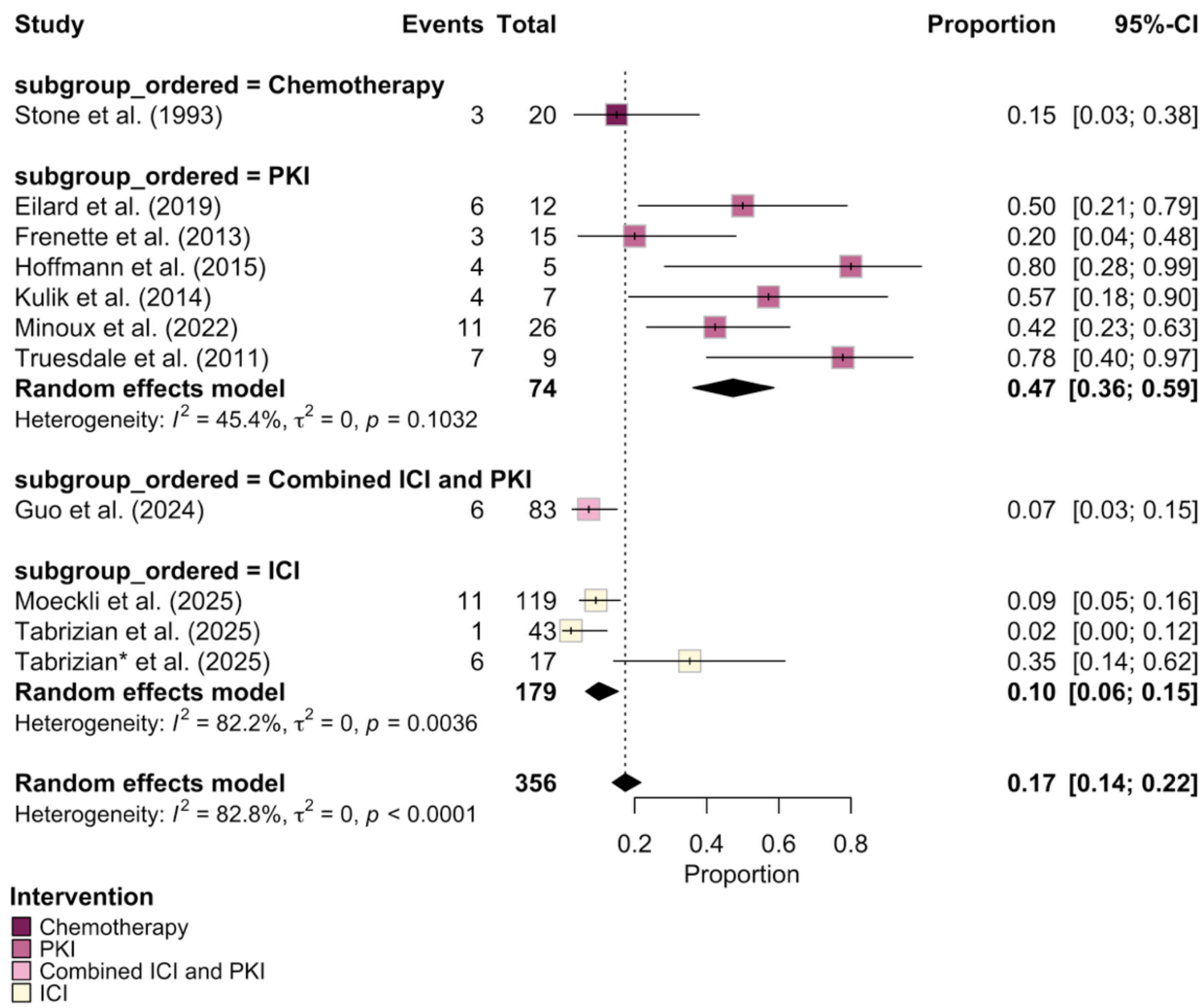

ESM 14 Subgroup-specific forest plot of pooled major postoperative complications rates. It illustrates the pooled estimates within each subgroup, previously defined by the systemic therapy given prior to liver transplant. Statistical analysis: Pooled estimates were derived using a fixed-effect model; any comparison across subgroups is statistically unsound and must not be made. Heterogeneity was assessed using the I^2 statistics, T^2 and Cochran's Q X^2 test. Significance threshold was set at p<0.05.

ESM 15: Scatter plot representing mortality proportion over median follow-up

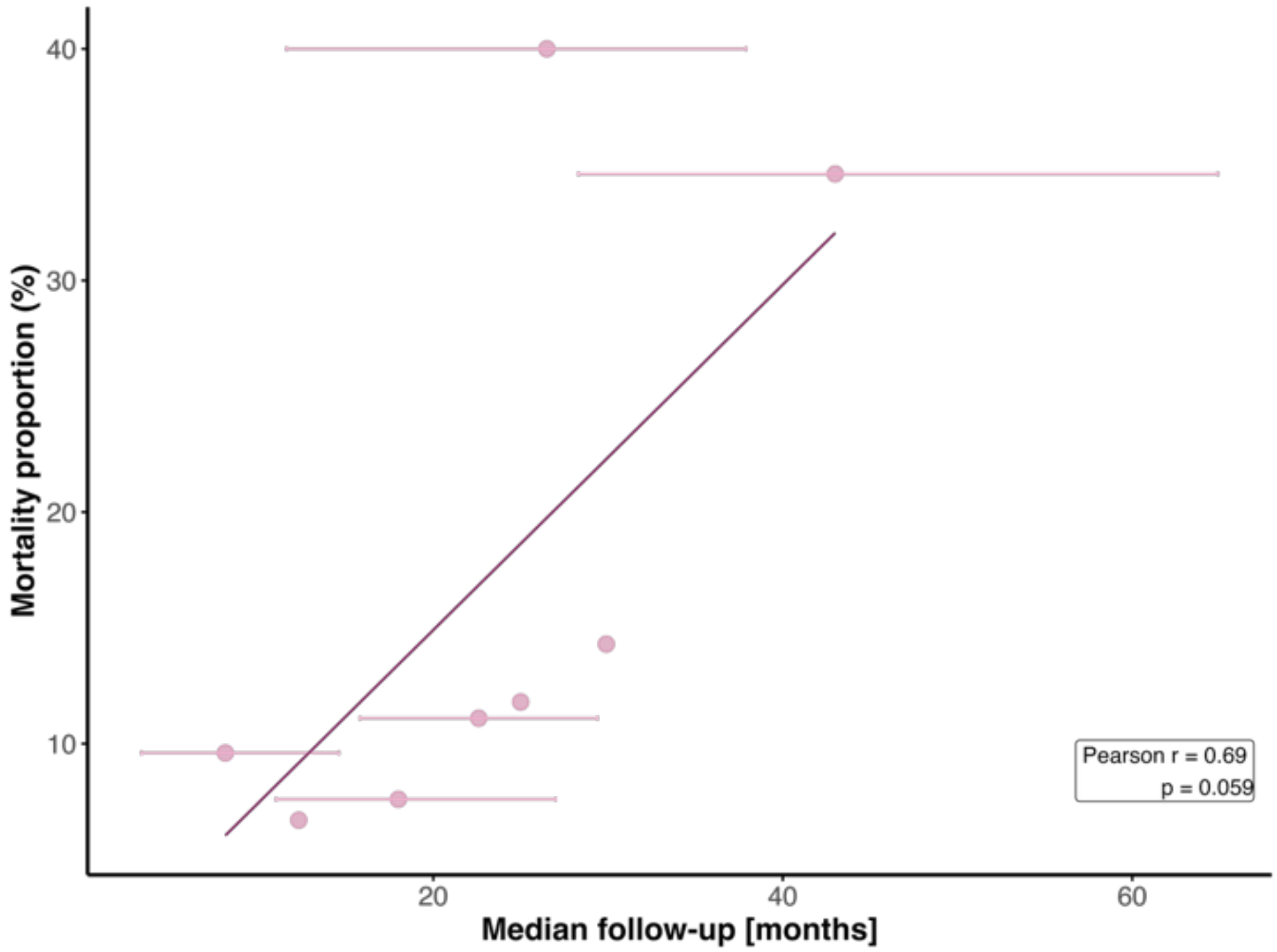

ESM 15 The scatter plot illustrates the relationship between mortality proportion and median follow-up duration across studies. Each point represents an individual study; horizontal bars indicate interquartile ranges. A fitted linear trend line illustrates a positive association; however, Pearson's *r* indicated no significant linear correlation. Significance threshold was set at $p<0.05$.

References

[1] Page MJ, McKenzie JE, Bossuyt PM, et al. The PRISMA 2020 statement: an updated guideline for reporting systematic reviews 2021. https://doi.org/10.1136/bmj.n71.

[2] Wan X, Wang W, Liu J, et al. Estimating the sample mean and standard deviation from the sample size, median, range and/or interquartile range. BMC Med Res Methodol 2014;14:135. https://doi.org/10.1186/1471-2288-14-135.

[3] Hozo SP, Djulbegovic B, Hozo I. Estimating the mean and variance from the median, range, and the size of a sample. BMC Medical Research Methodology 2005;5:13. https://doi.org/10.1186/1471-2288-5-13.
